# Automated assessment of technological and financial drivers of greenhouse gas reduction in sustainable renewable energy systems

**DOI:** 10.1038/s41598-026-40170-w

**Published:** 2026-02-21

**Authors:** Subhash Chandra, Ali Raqee Abdulhadi, Rouya Hdeib, N. Beemkumar, Abinash Mahapatro, Ashwin Jacob, Marwea Al-hedrewy, Temur Eshchanov, Bekzod Madaminov

**Affiliations:** 1 Department of Electrical Engineering, GLA University, Mathura, 281406 India; 2 Mechanical Engineering Department, University: Al-Turath University, Baghdad, 10013 Iraq; 3 College of Engineering, Applied Science University, Al Eker, Kingdom of Bahrain; 4 Department of Mechanical Engineering, School of Engineering and Technology, JAIN (Deemed to Be University), Bangalore, Karnataka India; 5 Department of Mechanical Engineering, Siksha ‘O’ Anusandhan (Deemed to Be University), Bhubaneswar, Odisha 751030 India; 6 Department of Mechanical Engineering, Sathyabama Institute of Science and Technology, Chennai, Tamil Nadu India; 7 College of Technical Engineering, The Islamic University, Najaf, Iraq; 8 College of Technical Engineering, The Islamic University of Al Diwaniyah, Al Diwaniyah, Iraq; 9 Urgench State University Named After Abu Rayhon Beruni, Urgench, Uzbekistan; 10 Department of General Professional Sciences, Mamun University, Khiva, Khorezm Uzbekistan

**Keywords:** Renewable energy, Emission reduction, Feature selection, Sensitivity analysis, Cross-validation, Energy storage efficiency, Energy science and technology, Engineering, Environmental sciences, Environmental social sciences

## Abstract

This study analyzes the capacity of renewable energy facilities to reduce greenhouse gas emissions using feature-based analysis approaches. The main goal is to identify the technological, economic, and environmental elements that most substantially influence emission reduction, serving as a basis for strategic planning and policy development. The dataset includes multiple renewable energy sources and financial variables. Predictive modeling was conducted via CatBoost Regression (CAT R) and Random Forest Regression (RFR), along with hybrid optimization via Transit Search Optimization (TSP) and Arithmetic Optimization Algorithm (AOA). Among the assessed configurations, the CAAO configuration not only achieved the highest predictive performance but also converged faster, demonstrating computational efficiency advantageous for real-time and large-scale energy planning. Feature analysis utilizing SHAP values, K-fold cross-validation, and sensitivity evaluation via the FAST method revealed that energy storage efficiency is the predominant factor, followed by financial incentives, underscoring the significance of both technological and economic aspects in emission reduction strategies. These findings offer an initial investigation and pragmatic suggestions rather than conclusive determinations. The findings indicate that feature-oriented assessments, when integrated with sophisticated predictive modeling, may substantially improve renewable energy planning and facilitate the formulation of context-specific, low-carbon policies. Importantly, by jointly employing variance-based global sensitivity analysis (FAST) and explainable machine learning (SHAP), the study reconciles an apparent discrepancy between structural system drivers (e.g., energy storage capacity) and predictive policy drivers (e.g., financial incentives). This dual-perspective analysis demonstrates that while storage dominates the physical response of emission reduction, incentive mechanisms primarily govern short-term predictive variability, offering a nuanced interpretability framework rarely achieved by single-method studies.

## Introduction

### Background

The alarming reduction of global fossil fuel reserves and the swift rise in greenhouse gas (GHG) emissions have emerged as significant worldwide issues, impacting economic stability, environmental integrity, and human welfare^1^. The energy industry accounts for over 40% of worldwide CO₂ emissions, whilst the transportation sector produces approximately 24%^2^. Anthropogenic CO₂ emissions disturb the carbon cycle, exacerbating climatic variability and resulting in severe weather phenomena, elevated sea levels, ecological degradation, biodiversity decline, and food insecurity^3^. Confronting these difficulties necessitates technically sound and sustainable ways that concurrently diminish emissions and uphold economic development^4^.

Renewable energy sources (RESs) have demonstrated efficacy in decarbonization and sustainable energy provision. Essential technologies encompass solar photovoltaics, wind turbines, hydroelectric systems, geothermal plants, biomass, tidal, and wave energy, all of which provide clean, plentiful, and sustainable substitutes for fossil fuels^5^. Incorporating renewable energy sources into electricity systems diminishes carbon emissions and air pollutants, while improving energy security, stabilizing supply, generating employment opportunities, and promoting technical innovation^6,7^. Furthermore, successful implementation necessitates consideration of storage capacity, efficiency, and grid interconnection to guarantee dependable and uninterrupted power supply^8^. The widespread implementation of renewable systems encounters practical obstacles, such as substantial initial capital expenditure, regulatory impediments, technology limits in storage and transmission, and site-specific environmental limitations^9,10^. These obstacles underscore the necessity for thorough planning, policy endorsement, and technical refinement to facilitate an efficient and sustainable energy transition^11^.

### Related works

Considerable research has addressed greenhouse gas (GHG) emissions across energy-intensive sectors, emphasizing the need for accurate quantification and prediction to inform mitigation strategies^12–14^. While earlier studies in transportation and industrial domains have advanced emission inventory methodologies—particularly through data-driven, bottom-up approaches that integrate multi-source operational data—their primary contribution lies in demonstrating the value of high-resolution, multivariate datasets for emission estimation and forecasting rather than sector-specific outcomes^15–18^.

In addition to reducing marine emissions, renewable energy systems have been thoroughly examined for their contribution to overall carbon mitigation. Wang^19^ showed that implementing renewable energy in China, using optimal provincial clean energy combinations, markedly decreases emissions. Abidi et al.^20^ emphasized the significance of policy formulation and regional customization for the successful adoption of renewable energy. Heshmati^21^ showed that technological progress, governance, and market rules improve the effectiveness of renewable energy in the EU-15 countries. Kumi et al.^22^ showed that elevating renewable energy penetration to 30% in Ghana might decrease emissions from 52,545.2 MtCO₂eq to 38,332.6 MtCO₂eq. Research conducted in Bangladesh by Dulal et al.^23^ highlighted the enduring advantages dependent on the expansion of renewable capacity in conjunction with supplementary carbon mitigation strategies. Kurte et al.^24^ established that over a 50% decrease in emissions is attainable with negligible cost increases using multi-objective optimization, whereas Marouani et al.^25^ projected that renewable energy may eradicate up to 90% of power-generating emissions by 2050. These studies emphasize that renewable energy is essential for decarbonization, dependent on strategic distribution, strong regulations, technical advancement, and integration with economic and environmental systems.

Despite these advances, most existing studies focus on policy evaluation, scenario-based projections, or sector-specific analyses, rather than developing integrated, data-driven predictive frameworks that simultaneously account for technical (capacity, storage), economic (financial incentives, funding mechanisms), and environmental (emission and pollution indicators) variables. Moreover, most works rely on aggregated or long-term projections, limiting their applicability for short- to medium-term planning and operational decision support.

#### Research gaps

Although substantial evidence supports the potential of renewable energy systems to mitigate greenhouse gas emissions, existing studies predominantly focus on policy effectiveness, regional deployment, or scenario-based assessments. While these approaches provide valuable macro-level insights, they often lack a cohesive analytical framework that can simultaneously integrate installed capacity, energy production and consumption, storage efficiency, financial incentives, and socio-economic outcomes within a unified predictive framework. Consequently, many investigations examine technological, economic, or policy variables in isolation, overlooking the nonlinear and interdependent mechanisms by which these factors collectively influence emission-reduction outcomes.

From a methodological standpoint, most prior works rely on traditional regression-based or single-model machine learning approaches, which are constrained by fixed model structures and limited capacity to adapt to heterogeneous data types and complex interaction effects. Variables such as installed capacity and storage efficiency exhibit continuous, nonlinear technical behavior, whereas financial incentives, funding sources, and grid integration levels introduce categorical, threshold-driven, and policy-induced discontinuities. Modeling these mixed characteristics with standard regression formulations can yield suboptimal representations of the underlying system dynamics.

Hybrid optimization–assisted machine learning frameworks offer a theoretically robust alternative to address this limitation. By integrating ensemble learners (e.g., CatBoost) with metaheuristic optimization algorithms (e.g., the Arithmetic Optimization Algorithm), such frameworks can simultaneously optimize model structure, feature interactions, and hyperparameter configurations. This enables more effective navigation of high-dimensional, non-convex solution spaces that arise when technological performance indicators interact with economic incentives and policy mechanisms. Unlike conventional models, hybrid approaches can adaptively balance exploration and exploitation during training, reducing susceptibility to local optima and improving robustness under diverse operating conditions.

Additionally, much of the existing literature emphasizes long-term projections, often neglecting short- and medium-term trade-offs where renewable energy deployment may yield delayed or uneven emission reductions due to storage inefficiencies or grid integration constraints. Empirical modeling of these transitional dynamics—particularly the role of energy storage and financial incentives in stabilizing emission benefits—remains limited.

Accordingly, there is a clear need for data-driven, multi-variable modeling frameworks that can capture the aggregated environmental, financial, and socio-economic impacts of renewable energy systems while explicitly accounting for nonlinear interactions and optimization uncertainty. Addressing this gap can enhance predictive reliability, support informed investment decisions, and strengthen evidence-based policymaking for sustainable energy transitions.

### Objective

The main aim of this research is to investigate the principal factors influencing greenhouse gas emission reduction in renewable energy systems, emphasizing specific features. Notwithstanding the increasing global implementation of renewable energy, prior research has predominantly focused on system-level results, including overall energy production or general emission reductions, thereby creating a knowledge deficit concerning the comparative impact of specific technical, financial, and environmental variables. This gap restricts the ability to make educated, evidence-based decisions that enhance renewable energy deployment for emission reduction. This study addresses the gap by highlighting the influence of factors such as energy storage capacity and efficiency, installed capacity, energy production and consumption, grid integration level, initial investment, funding sources, financial incentives, and air pollution reduction, to determine which elements most significantly affect emission outcomes.

The study utilizes CatBoost Regression (CATR) and Random Forest Regression (RFR) as predictive models, enhanced by hybrid optimization techniques including Transit Search Optimization (TSP) and Arithmetic Optimization Algorithm (AOA). This combination permits simulation of intricate, nonlinear relationships between technological, financial, and environmental variables, yielding a higher resolution view of feature-level impacts upon emissions reductions. The research provides initial explorations of the influence of particular technical and economic factors upon emission reduction by marrying feature sensitivity analysis with strong regression and optimization techniques. These insights provide a basis for further research and practical evaluations, directing initiatives to enhance the efficacy of renewable energy implementation, while recognizing that the findings are exploratory and meant as a recommended framework rather than definitive proof. Unlike many ensemble learning and metaheuristic-tuned frameworks that incur high computational costs, the proposed CAAO model is designed to balance predictive performance with computational efficiency. By employing a compact optimizer–model coupling and a bounded search space, the framework reduces training time and optimization overhead, which is particularly advantageous for iterative policy analysis and real-time sustainability planning scenarios.

In addition to greenhouse gas mitigation, the study considers air pollution reduction as a co-benefit indicator reflecting localized improvements in environmental quality. The Air Pollution Reduction Index (APRI) is a normalized composite measure of reductions in conventional air pollutants (such as SO_2_, NO_x_, and particulate matter) associated with renewable energy deployment, energy efficiency gains, and reduced reliance on fossil fuels. Importantly, this index is treated as an independent explanatory variable, capturing complementary but not redundant environmental effects relative to greenhouse gas emission reduction.

**Fig. 1:**
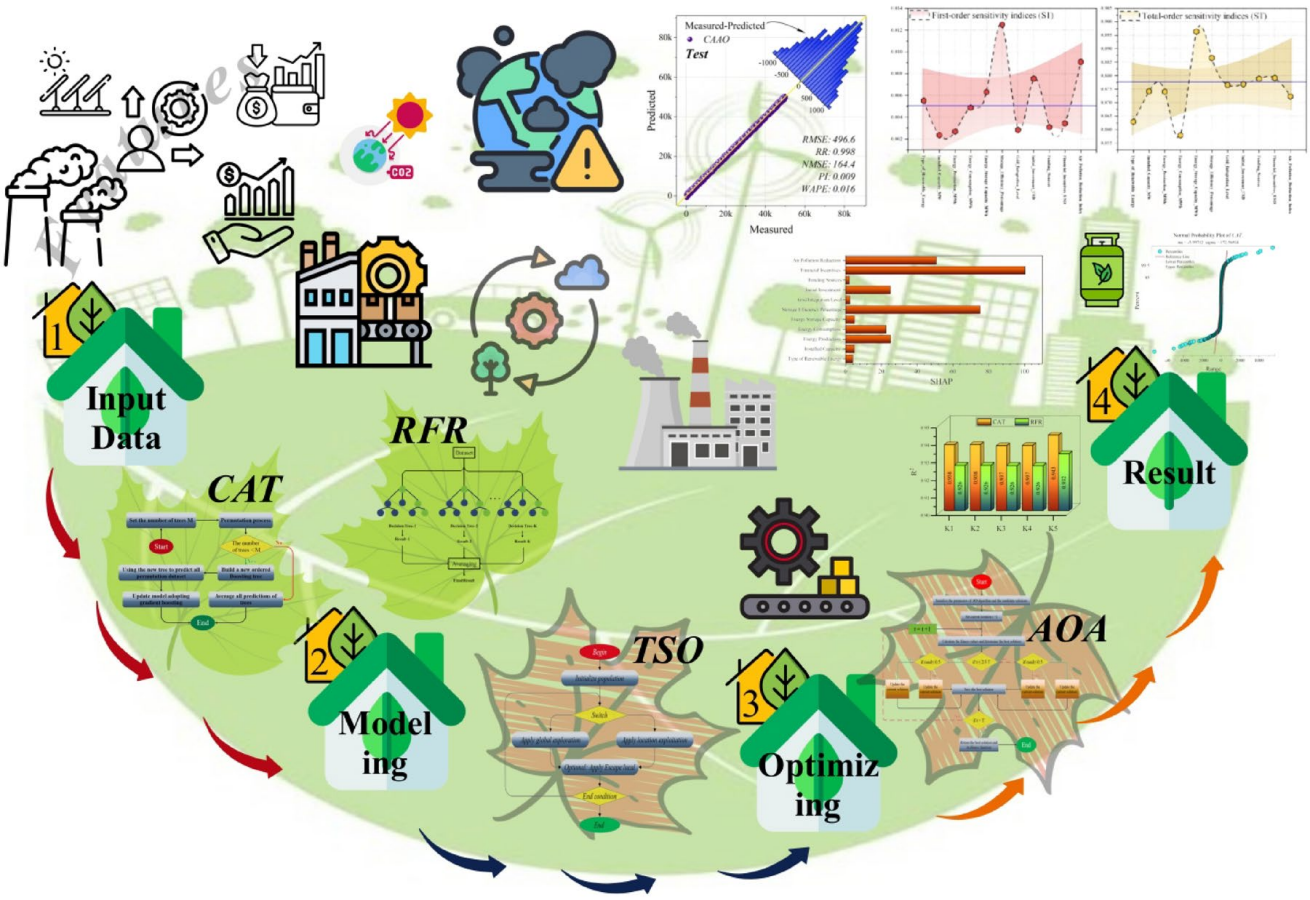
Overview of the study process.

It should be noted that this study is not intended as a country-specific or regionally calibrated policy assessment. Instead, the analysis is based on an aggregated renewable energy dataset compiled from publicly available sources, designed to represent plausible ranges and internally consistent relationships among technological, financial, and environmental variables commonly observed in renewable energy systems. The primary objective is therefore to investigate relative feature importance, interaction effects, and modeling behavior, rather than to produce directly transferable national policy prescriptions. This framing allows the proposed framework to serve as an exploratory and methodological reference for future, region-specific studies. Figure 1 presents overview of the study process.

## Data description

Before predictive modeling, the renewable energy dataset obtained from Kaggle underwent thorough preparation to guarantee data quality, consistency, and appropriateness for machine learning techniques (https://www.kaggle.com/datasets/girumwondemagegn/dataset-for-renewable-energy-systems). The dataset contains 15,000 samples which variables of the dataset presented in Table 1. Initially, absent values were detected and addressed by suitable imputation methods, including mean imputation for numerical variables (installed capacity, yearly energy output) and mode imputation for categorical features (energy type categorization). Outliers were identified using statistical methods, including Z-score and interquartile range (IQR), and were either rectified or eliminated to avert bias in the prediction models. Furthermore, feature selection methodologies, such as correlation analysis and variance thresholding, were employed to minimize redundancy and preserve the most useful variables for modeling greenhouse gas emission reduction. The preparation approach involved dividing the dataset into training and testing groups, usually in an 80:20 ratio, to assess model generalization.

**Table 1:** Summary of input and output variables used in the study.

Variable	Description
Type_of_Renewable_Energy	Solar, Wind, Hydro, Geothermal, Biomass, Tidal, Wave
Installed_Capacity_MW	Installed capacity of the system
Energy_Production_MWh	Yearly energy production
Energy_Consumption_MWh	Yearly energy consumption
Energy_Storage_Capacity_MWh	Energy storage capacity
Storage_Efficiency_Percentage	Efficiency of the energy storage system
Grid_Integration_Level	Level of grid integration (Fully Integrated to Isolated Microgrid)
Initial_Investment_USD	Project investment cost
Funding_Sources	Source of funding (Government, Private, Public–Private Partnership)
Financial_Incentives_USD	Monetary incentives for the project
Air_Pollution_Reduction_Index	Air pollution reduction indicator
Jobs_Created	Number of jobs generated
GHG_Emission_Reduction_tCO2e	Reduction in greenhouse gas emissions

The target variable, GHG_Emission_Reduction (tCO₂e), represents the estimated reduction in greenhouse gas emissions associated with renewable energy system deployment. This variable is treated as an outcome indicator and is not directly calculated from any single input feature within the dataset. However, it may exhibit statistical association with several explanatory variables that reflect shared technological or policy drivers (e.g., energy production levels, environmental co-benefits). Accordingly, additional analyses were conducted to assess potential information leakage.

The APRI is a dimensionless composite indicator that reflects the relative reduction in local air pollutants attributable to the operation of a renewable energy system. The index aggregates normalized reductions in key pollutants commonly associated with fossil-based generation (e.g., sulfur dioxide, nitrogen oxides, and particulate matter), based on representative emission-factor ranges reported in energy and environmental assessment studies. The index is not directly calculated from greenhouse gas emissions; instead, it serves as a co-benefit metric that captures near-term, location-specific environmental improvements that may occur independently of total CO₂-equivalent reductions.

**Fig. 2:**
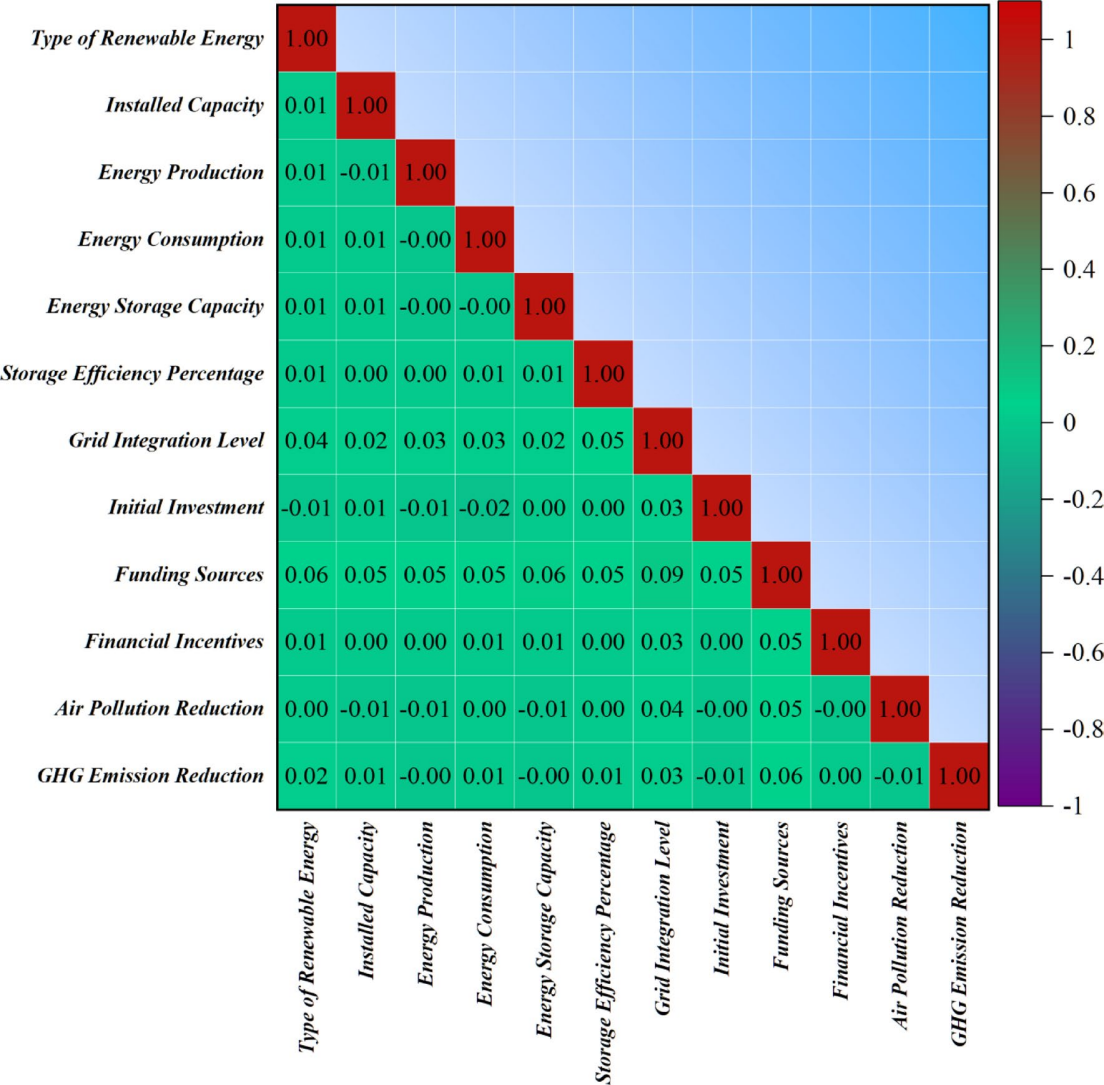
Relationships between input and output variables with the correlation matrix.

Figure 2 illustrates the pairwise correlations between input variables and the greenhouse gas emission reduction target. Higher correlations observed for variables such as storage efficiency and installed capacity reflect their substantive influence on system performance and emission avoidance potential, rather than direct computational dependence. Lower correlations indicate weaker marginal contributions. Although certain variables—such as energy production and the air pollution reduction index—exhibit moderate to strong associations with the target, these relationships arise from shared underlying drivers (e.g., displacement of fossil generation and improved system efficiency), not from circular construction. To mitigate potential target leakage, correlation analysis was complemented with feature ablation experiments, ensuring that predictive performance is not dominated by any single proxy variable. To assess potential target leakage, additional experiments were conducted in which high-risk variables including the Air Pollution Reduction Index were removed from the feature set. The CAAO model maintained strong predictive accuracy under this reduced-input configuration, with only a moderate decline in performance metrics. This confirms that the near-perfect performance observed in the full model arises from distributed multivariate learning and interaction effects, rather than trivial reproduction of the target variable. These findings indicate that the model generalizes beyond any single correlated feature and that the reported performance reflects genuine predictive structure within the data.

**Fig. 3:**
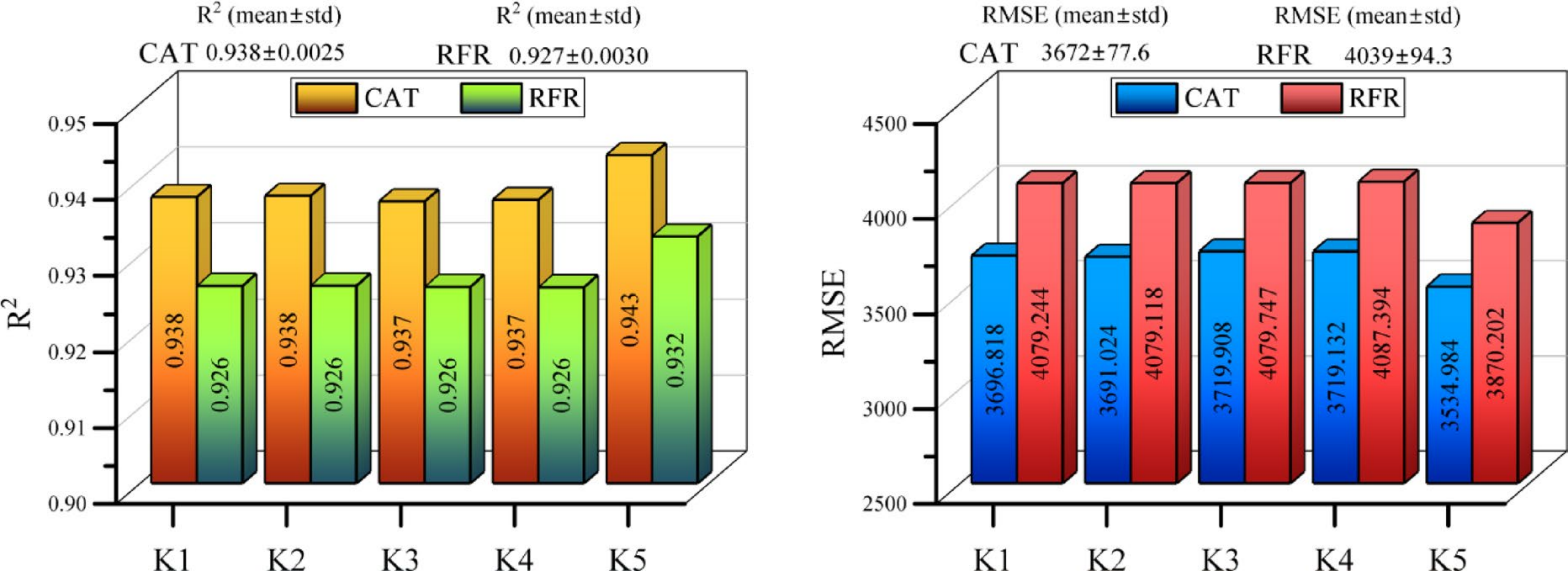
Performance metrics (R^2^ and RMSE) for each model, reported as mean ± standard deviation across five cross-validation folds.

Figure 3 presents the results of the K-fold cross-validation procedure implemented within the selected predictive framework, illustrating model performance across five validation folds. This study is not intended for direct comparison between different predictive models. Instead, cross-validation employed to evaluate robustness and consistency of model behavior across alternative data partitions and to identify representative folds for subsequent feature analysis. The reported R^2^ and RMSE values indicate the proportion of variance explained in each fold, offering insight into the model’s generalization performance across varying training–validation configurations. Folds exhibiting higher explained variance correspond to data partitions with balanced feature distributions and reduced sampling bias, and are therefore considered more representative for interpretability and sensitivity analyses. Conversely, folds with lower explained variance reflect inherent variability within the dataset and are retained to ensure a comprehensive robustness assessment. Importantly, the purpose of this analysis is not to rank or declare superiority among models, but to ensure that feature relationships identified through correlation analysis, FAST sensitivity analysis, and SHAP explainability are derived from stable and reliable data configurations. By emphasizing fold-level consistency rather than inter-model ranking, the K-fold framework supports robust interpretation of the technological, economic, and environmental drivers of greenhouse gas emission reduction.

Table 2 presents a detailed summary of the input features and output variable, encompassing their statistical characteristics and elucidating the variability and distribution of data pertinent to renewable energy emission reduction modeling. The input features include technical, financial, and environmental parameters, including renewable energy type, installed capacity, energy production and consumption, storage capacity and efficiency, grid integration level, initial investment, funding sources, financial incentives, and air pollution reduction. The statistical characteristic analysis indicates that there is high variability throughout the characteristics. The installed capacity, energy output and energy consumption have high variability, illustrating the variability of renewable energy systems and scope of operations. The storage-associated characteristics, including efficiency and capacity, have high variability, indicating different technical performances of systems. The economic and financial inputs, including incentives and startup capital, have high variability, illustrating different economic states of renewable energy installation. The environmental aspects, including reduction in air pollution, have high variability, illustrating the different influences of renewable energy projects. The result variable, GHG emission reduction, reflects the variability seen in input characteristics, highlighting the intricacy of the prediction process. The statistical features highlight the need for strong prediction models that can manage varied and nonlinear connections across technological, economic, and environmental elements to properly estimate emission reductions in renewable energy systems.

**Table 2:** Overview of input features and output variables with their statistical properties.

Variables	Unit	Data Role	Characteristics				
			Max	Min	Median	Average	St. Dev
Type of Renewable Energy		Inputs	7	1	4	3.97	2
Installed Capacity	MW		1000	1.09	493	496	288
Energy Production	MWh		5.00E + 05	1030	2.53E + 05	2.52E + 05	1.44E + 05
Energy Consumption	MWh		4.50E + 05	584	2.25E + 05	2.26E + 05	1.29E + 05
Energy Storage Capacity	MWh		1.00E + 04	2.2	5050	5030	2890
Storage Efficiency Percentage	%		100	50	75.3	75.2	14.5
Grid_Integration Level	–		4	1	3	2.5	1.12
Initial Investment	USD		5.00E + 08	1.01E + 06	2.54E + 08	2.51E + 08	1.43E + 08
Funding Sources	–		3	1	2	2	0.817
Financial Incentives	USD		2.00E + 07	5.16E + 04	1.00E + 07	1.00E + 07	5.79E + 06
Air Pollution Reduction Index	–		100	1.01	50.3	50.7	28.6
GHG Emission Reduction	tCO₂e	Output	50,000	101	25,400	25,200	14,400

### Dataset source and characteristics

The renewable energy dataset used in this study was obtained from the Kaggle open-data platform, which hosts a variety of datasets compiled for research, education, and benchmarking. The selected dataset is a synthetic, aggregated representation of renewable energy systems, constructed to reflect realistic operational, financial, and environmental characteristics reported in publicly available renewable energy project documentation, energy outlook reports, and feasibility studies.

Importantly, the dataset does not correspond to a single geographic region or country, nor does it represent direct measurements from a specific national energy system. Instead, it integrates plausible ranges and internally consistent relationships across multiple renewable energy technologies, including solar, wind, hydroelectric, geothermal, biomass, tidal, and wave energy systems. This design enables comparative analysis across technologies while avoiding regional bias.

The primary motivation for adopting this dataset is its holistic variable composition, which simultaneously captures:

- Technological attributes (e.g., installed capacity, energy production, storage capacity, and storage efficiency),
- Economic and financial factors (e.g., initial investment, funding sources, and fiscal incentives), and
- Environmental and socio-economic outcomes (e.g., greenhouse gas emission reduction, air pollution reduction index, and jobs created).

Such a combination is rarely available at a sufficient scale in real-world datasets, particularly for studies focusing on feature importance, sensitivity analysis, and hybrid machine learning optimization. Therefore, the dataset is well-suited to the exploratory and methodological objectives of this research.

## Methodology

### Random forest regression (RFR)

Numerous decision trees utilized as independent regression models are developed employing the random forest methodology. The outcome of the RF regression is determined by the average of the outputs produced by several decision trees, ranging from a few hundred to thousands. The Classification and Regression Tree (CART), sometimes referred to as the Decision Tree, was initially introduced by Breiman et al.^26,27^. The extent of a tree’s learning capacity is contingent upon the intricacy of the data provided to it. Decision nodes and terminal nodes constitute any decision tree. The extent of a tree’s learning capacity is contingent upon the intricacy of the data provided to it. Decision nodes and terminal nodes constitute any decision tree. The input vector X, including $m$ characteristics, may be expressed as $X=\left\{{x}_{1},{x}_{2},\dots ,{x}_{m}\right\}$. The scalar output is denoted by the symbol Y. The formal representation of ${R}_{n}$, denoting the set of n observations constituting the training set, is given by Eq. (1).

1 $${R}_{n}=\left\{\left({X}_{1},{Y}_{1}\right), \left({X}_{2},{Y}_{2}\right), \dots , \left({X}_{n},{Y}_{n}\right)\right\}, X\in {R}^{m}, Y\in R.$$

In the training phase, the method partitions the input data at each node, enabling the adjustment of split function parameters to achieve an optimal alignment with the ${R}_{n}$ set. In the initial phase of the inquiry, a decision tree must be employed to select the most advantageous segmentation among all variables. The primary node initiates the partitioning process, with each subsequent node dividing the incoming dataset X based on its designated split strategy. The previously described technique is executed recursively until no more nodes or branches can be accessed. A node or branch may be designated as a tree leaf or terminal node.

In arboriculture, it is customary to restrict a tree’s development upon reaching a certain threshold or when a designated node has a predefined number of observations beneath a threshold value. To forecast the outcomes of $H=\left(X,{\Theta }_{K}\right)$, a predictive function representing $\widehat{H}=\left(X,{R}_{n}\right)$ is created following the requisite training process. L tree-structured base classifiers $H=\left(X,{\Theta }_{K}\right)$ are employed for RFR, where $K=1,\dots ,L$ and ${\Theta }_{K}$ denotes a set of independently distributed random vectors. The RF methodology employs a random selection of a subset of features or a segment of the training dataset for each decision tree. In random sampling, sometimes referred to as "bootstrap," n observations are selected randomly from ${R}_{n}$ to facilitate repeated selection, with each observation having an equal probability of selection, namely $1/n$. In the bagging approach, a selection of bootstrap samples $\left({S}_{n}^{{\Theta }_{1}}, \dots , {S}_{n}^{{\Theta }_{q}}\right)$ is made, followed by the application of the previously outlined decision tree technique. This leads to the formation of a set of $q$ prognostic trees, or $\widehat{h}\left(X, {S}_{n}^{{\Theta }_{1}}\right), \dots , \widehat{h}\left(X, {S}_{n}^{{\Theta }_{q}}\right)$. One stage in the aggregation process is determining the mean value of the outputs generated by each tree. Consequently, it can be said that the expected value of $Y$(denoted as ${Y}{\prime}$) may be derived using the methodology outlined in.

2 $$\widehat{Y}=\frac{1}{q}\sum_{l=1}^{q}{\widehat{Y}}_{l}=\frac{1}{q}\sum_{l=1}^{q}\widehat{H}\left(X, {R}_{n}^{{\Theta }_{l}}\right)$$

**Fig. 4:**
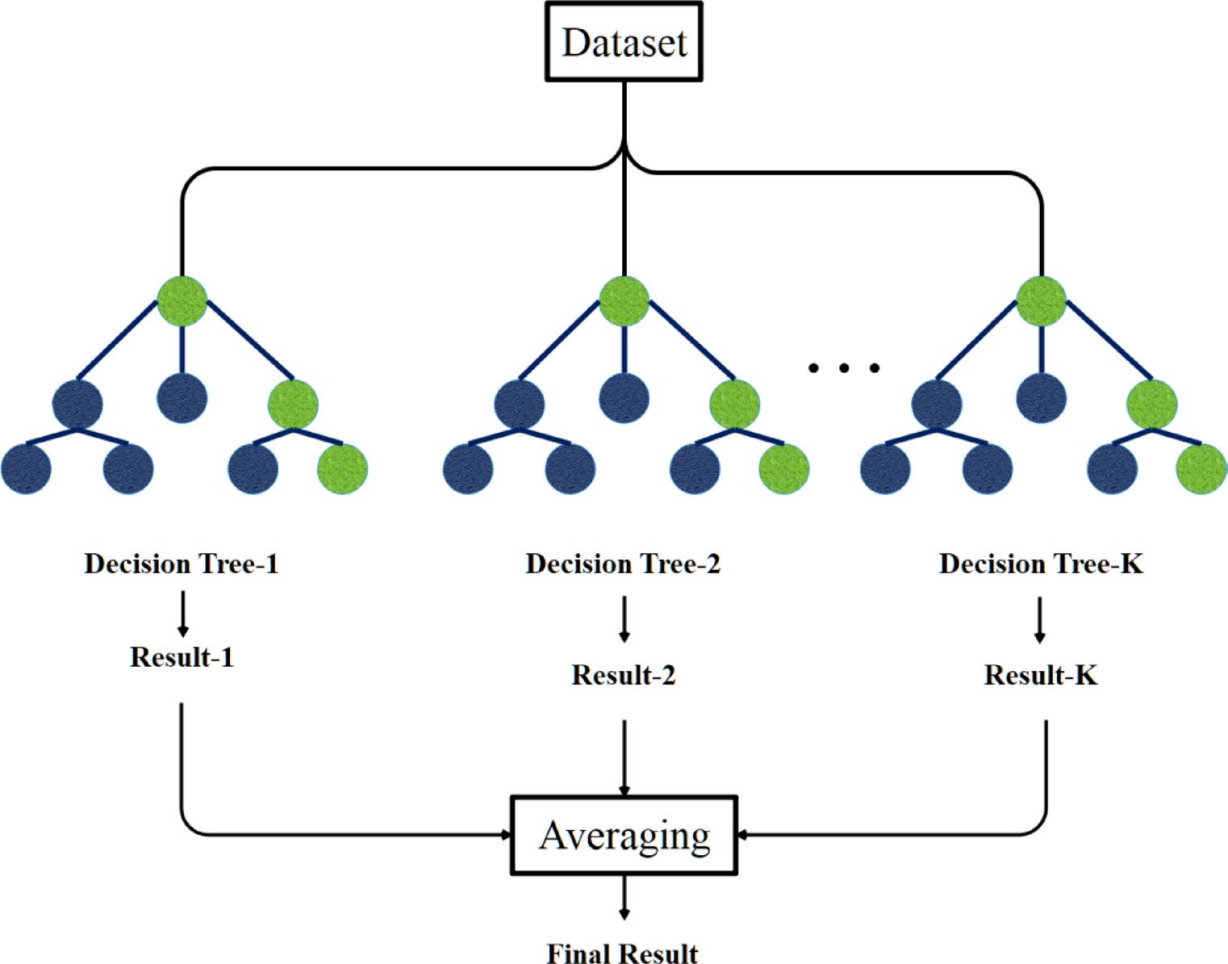
Structure of the RFR model.

In RFR on ${\widehat{Y}}_{l}$​, with some training data potentially used multiple times, while other samples may remain unused. This selective data utilization can influence learning efficiency. The bagging approach, which constructs trees using random bootstrap samples, enhances model stability and improves tolerance to minor irregularities in the input data. A key principle of RFR is the growth of unpruned trees, which ensures computational efficiency and robustness. RFR primarily requires tuning two parameters: the number of trees ${n}_{tree}$ and the number of randomly selected features per split ${(m}_{try})$. Increasing (${n}_{tree}$)​ generally improves prediction accuracy and model longevity, although it also increases computational cost. Beyond a certain point, additional trees provide diminishing returns due to convergence in generalization error, with ${n}_{tree}=500$ is often used as a baseline. The parameter ${m}_{try}$ can enhance individual tree performance but may also increase inter-tree correlation. The algorithm generates ${n}_{tree}$​ bootstrap datasets, each used to build an unpruned regression tree by randomly selecting ${m}_{try}$​ predictors at each split. Predictions are aggregated through regression averaging, improving overall reliability. Out-of-bag (OOB) samples allow error estimation and reduce overfitting, making RFR a stable, accurate, and user-friendly regression method. Figure 4 illustrates the workflow process of RFR.

### Cat boost regression (CATR)

Cat Boost Regression (CATR) is a gradient boosting technique optimized for the effective management of categorical data while reducing information loss. CAT R, proposed by Prokhorenkova et al.^28^ and subsequently refined by Dorogush et al.^29^, distinguishes itself from other gradient boosting techniques by employing ordered boosting, hence reducing target leakage and improving performance on small datasets. This method is especially appropriate for datasets including diverse feature types and constrained sample sizes.

**Fig. 5:**
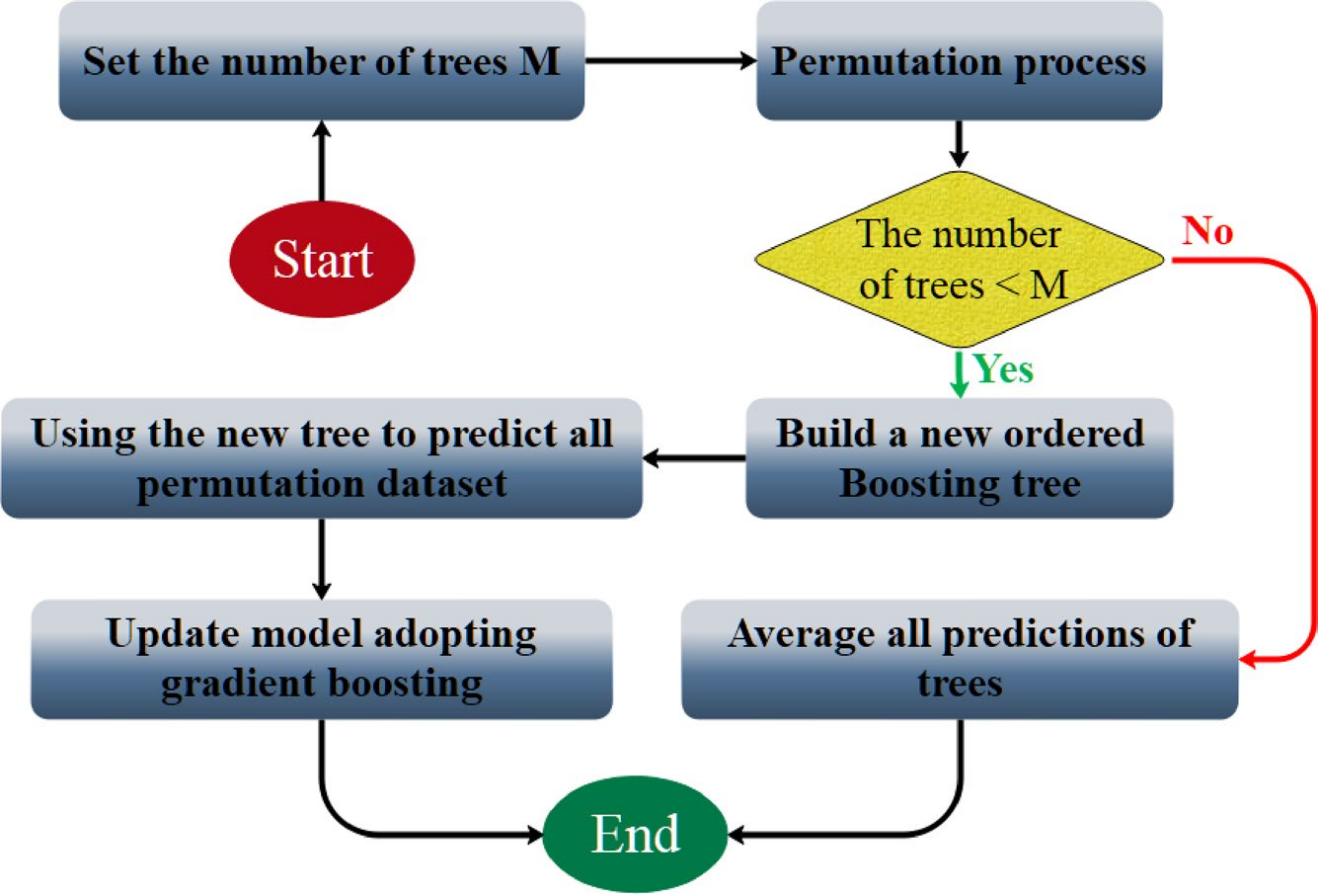
Flowchart of the CATR.

CATR processes categorical variables by converting them to numerical representations, often during preprocessing. Generally, each original categorical characteristic is substituted with one or more numerical encodings or binary components that correspond to each category, enabling the algorithm to analyze these variables without creating bias. The application of random permutations in leaf value estimation diminishes overfitting, a prevalent concern in conventional gradient boosting techniques. The essence of CATR is the binary decision tree, which divides the feature space into separate leaf regions *Rj*. The anticipated outcome for an input vector ${x}_{i}$. may be articulated as:

3 $$Z=H\left({x}_{i}\right)= \sum_{j=1}^{J}{c}_{j}{1}_{x\in {R}_{j}}$$

where ${R}_{j}$ denotes the discontinuous region associated with the $j-th$ leaf, ${c}_{j}$ is the leaf value and $H\left({x}_{i}\right)$ signifies the anticipated function for the input characteristics. Empirical research has established the efficacy of CATR across several data types, including tabular, time-series, and sector-specific datasets, for instance, in banking and finance. Its capacity to manage categorical features inherently, coupled with effective gradient boosting techniques, guarantees consistent and precise predictions while preventing overfitting. Thus, CATR is esteemed as a cutting-edge technique for regression problems with diverse datasets. Figure 5 presents the operational framework of the CatBoost model.

### Archimedes optimization algorithm (AOA)

The AOA is a population-based metaheuristic developed from the behavior of submerged objects in a fluid medium. In this context, each object symbolizes a possible solution, assigned starting random placements, accelerations, densities, and volumes. Like population-based approaches, AOA begins by initializing the original population and evaluating its fitness. AOA incrementally modifies the solutions until a termination criterion is satisfied. In each iteration, the volumes and densities of the objects are modified based on their distance from the currently optimal solution. It aids in guiding the search towards ideal areas of the search space. AOA employs a transfer operator (TF) that governs the ratio of exploration (finding new areas) to exploitation (enhancing current solutions). In the early rounds, the algorithm emphasizes exploration and gradually shifts towards exploitation as TF increases over iterations. A supplementary density-reduction factor facilitates convergence by progressively diminishing population variety.

**Fig. 6:**
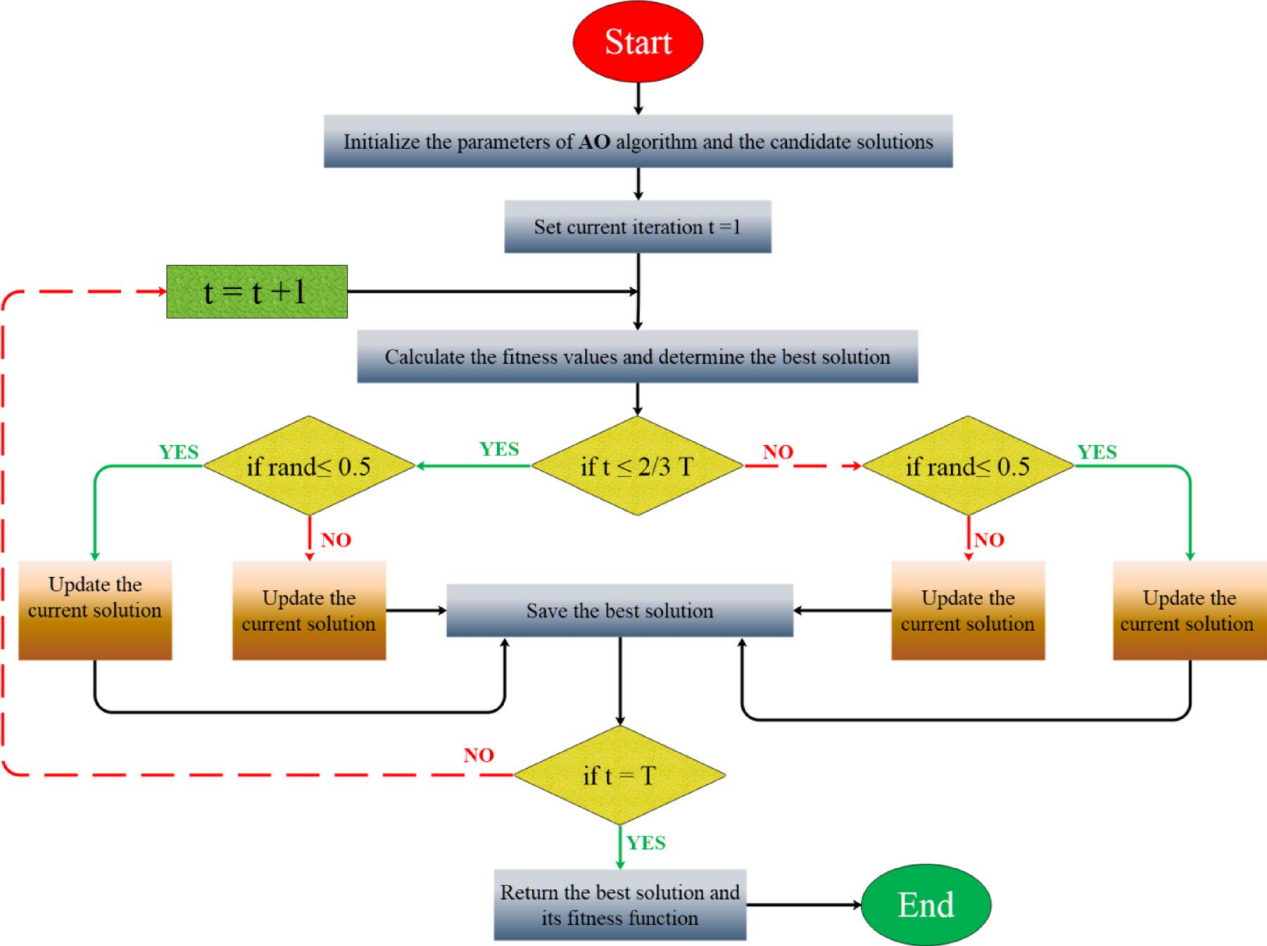
Flowchart of the AOA optimizer.

Their behavior depends on the occurrence of a “collision.” During the exploration phase (0.0 < TF < 0.5), objects simulate collisions by responding to randomly selected neighbors, adjusting their acceleration accordingly. During the exploitation phase (> 0.5), objects modify their placements based on the optimal knowledge obtained thus far. Normalized acceleration dictates their modes and directs their movement across the search space. The method modifies the location of each item based on its normalized acceleration and dynamic step-size parameter. It guarantees a substantial step size in the first phases (to facilitate broad exploration) and subsequently diminishes (to allow for more precise exploitation near optimal solutions). It possesses a direction flag that alters the update direction randomly to promote variety and prevent premature convergence. Finally, the fitness of the new population is evaluated, and the optimal solution is subsequently revised. It continues to repeat until the termination criterion is met, which may be either a limit number of iterations or an acceptable level of fitness. Effectively integrates global and local search algorithms through adaptive processes, rendering it suitable for addressing complex optimization challenges. For further information, consult^30^. Figure 6 depicts the operational flow of the AOA.

### Transit Search Optimization Algorithm (TSOA)

The quantity of host stars (ns) and the signal-to-noise ratio (SN), derived from the transit model, are two essential elements of the TSOA methodology. The noise is quantified by the standard deviation of data collected outside the transit period. The product of SN and *ns* establishes the starting population size for TS. This section examines the five essential phases of TSOA. The program initially picks a galaxy and a random center inside the search space and thereafter employs ${L}_{R}$ to examine ns × SN random patches to delineate the galaxy’s habitable zones, or life belts. The leading ns regions exhibiting the highest fitness, signifying a substantial probability of sustaining life, are then chosen for further algorithmic procedures^31^. During the first phase, accuracy criteria may encompass test sample errors, cross-validation, and substitution. Equation (4) indicates that the re-substitution error is derived from the identical dataset as the predictor p and is calculated as the mean squared error.

4 $$E\left(p\right)=\frac{1}{N}\sum_{i=1}^{N}{\left({u}_{i}-p\left({v}_{i}\right)\right)}^{2}$$

Recall that $\left({u}_{i},{v}_{i}\right)$ represents the learning samples for each $i=\mathrm{1,2},...,N.$ To assess the correlation error across samples, sample $X,$ including size $N$, is divided into $k$ subsamples, ${X}_{1},{X}_{2},\dots ,{X}_{k}$, each having approximately equivalent sizes ${N}_{1},{N}_{2},\dots , \,\,and \,\,{N}_{k}$, respectively. The subsample $X-{X}_{k}$ is utilized to generate the predictor p. Subsequently, as shown by Eq. (5), the cross-validation error is calculated utilizing the subsample ${X}_{k}$.

5 $${E}^{CV}\left(p\right)=\frac{1}{{N}_{k}}\sum_{k}\sum_{({u}_{i},{v}_{i})\in {X}_{k}}{\left({v}_{i}-{p}^{\left(k\right)}\left(u\right)\right)}^{2}$$

The subsample $X-{X}_{k}$ is utilized to obtain ${p}^{\left(k\right)}\left({u}_{i}\right)$. The test sample error divides the total occurrences into two subsamples, ${N}_{1}$ and ${N}_{2}$, with sizes $N$ and ${N}_{2}$, respectively. Equation (6) presents the outcome of the test sample error calculation.

6 $$E^{ts} \left( p \right) = \frac{1}{{N_{2} }}\mathop \sum \limits_{{\left( {u_{i} ,v_{i} } \right) \in X_{2} }}^{N} \left( {v_{i} - p\left( {u_{i} } \right)} \right)^{2}$$

When ${X}_{2}=Sub-sample$, no information is utilized to construct the predictor. The subsequent phase in the DTR-based methodology involves selecting the splits for predicting the values of the continuous dependent variable. The node impurity metric, which measures the relative homogeneity of instances within terminal nodes, is commonly employed to evaluate splits. Equation (7) illustrates the application of least-squares deviation for assessing a node’s impurity in a regression tree.

7 $$R\left(t\right)=\frac{1}{{N}_{W}\left(t\right)}\sum_{i\in t}{W}_{i}{f}_{i}{\left({u}_{i}-\overline{v }\left(t\right)\right)}^{2}$$

**Fig. 7:**
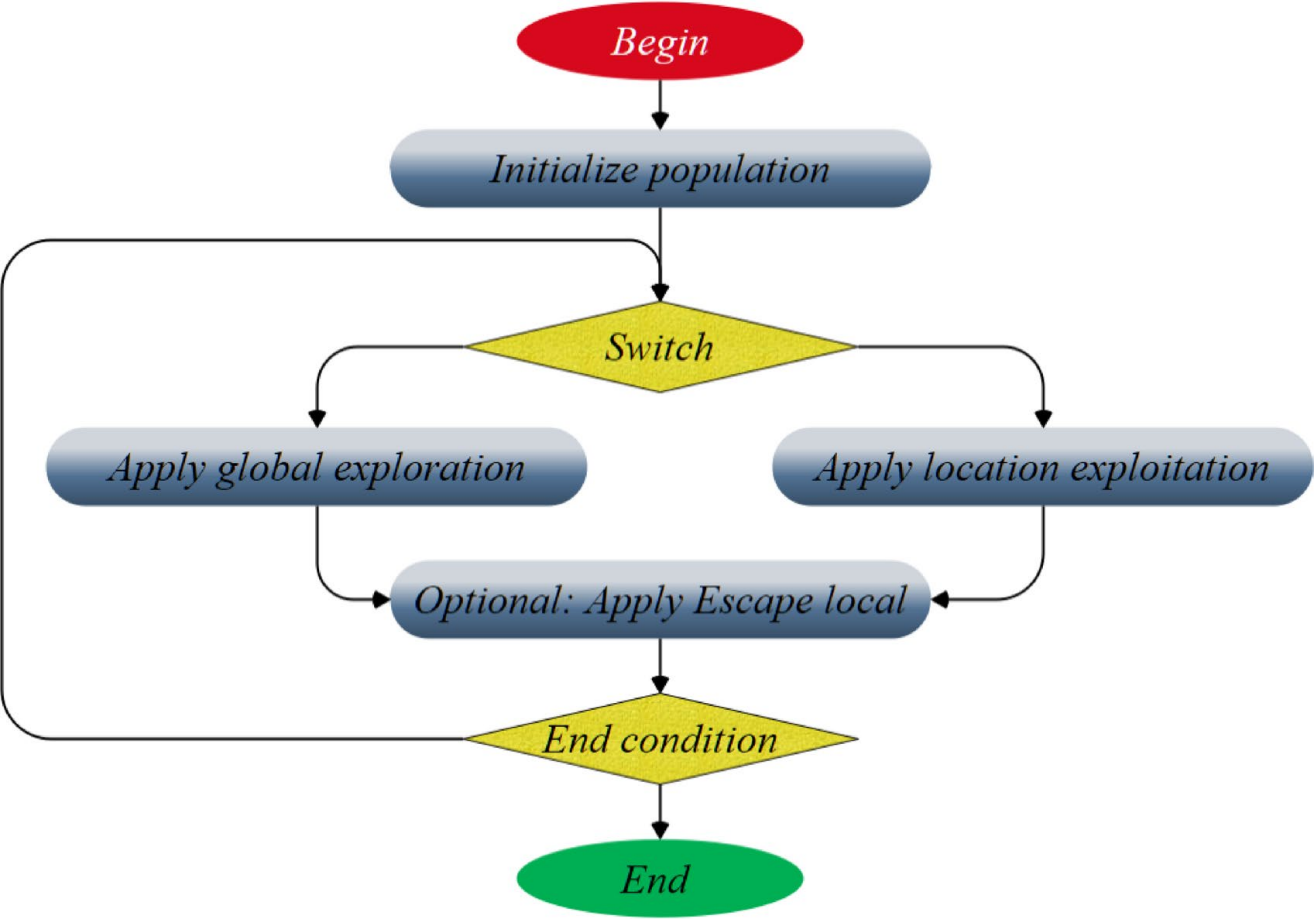
Flowchart of TSOA.

${N}_{w}\left(t\right)$ represents the weighted case count in node $t$. In scenario I, ${f}_{i}$ represents the frequency variable, whereas ${w}_{i}$ denotes the weighting variable. ${v}_{i}$ represents the value of the response variable. The weighted mean for node t is denoted as $\overline{v }\left(t\right)$. The third step employs the fewest nodes necessary to determine when to cease partitioning. The last phase in choosing the ideal tree frequently involves pruning it to the suitable dimensions^32^. The tree exhibiting the shortest dimensions and the least number of mistakes was pruned for the research. Figure 7 shows the functional framework of the TSOA algorithm.

### Rationale for selecting AOA and TSO hybrid optimizers

The hyperparameter optimization problem associated with ensemble learning models such as CatBoost and Random Forest is inherently non-convex, discontinuous, and high-dimensional, characterized by multiple local optima arising from tree depth, ensemble size, and regularization interactions. Classical gradient-based optimization methods are therefore unsuitable for this search space.

In this study, AOA and TSO were selected due to their complementary search behaviors and demonstrated robustness in complex, nonlinear optimization settings. AOA employs arithmetic operators to balance exploration and exploitation dynamically, enabling efficient traversal of rugged solution landscapes with reduced premature convergence compared to evolutionary operators commonly used in Genetic Algorithms (GA). In contrast, TSO simulates directed transition mechanisms that enhance global search capability while maintaining convergence stability, which is particularly advantageous for optimizing discrete and semi-continuous hyperparameters such as tree depth and ensemble size.

Compared to Particle Swarm Optimization (PSO), which may suffer from swarm stagnation in highly irregular objective spaces, and GA, which often requires extensive parameter tuning and higher computational cost, AOA and TSO provide parameter-efficient, computationally stable, and convergence-consistent optimization strategies. These characteristics make them well suited for the non-convex hyperparameter landscapes encountered in renewable energy emission reduction modeling, where predictive accuracy and computational efficiency are both critical.

Accordingly, the use of AOA and TSO is motivated not by algorithmic novelty alone, but by their theoretical compatibility with non-convex search spaces, limited tuning requirements, and suitability for hybrid integration with tree-based ensemble models.

### Performance evaluators

This study uses performance evaluators to measure the prediction accuracy and reliability of emission reduction models. Root Mean Square Error (RMSE) quantifies the average magnitude of prediction mistakes, attributing greater significance to bigger discrepancies. The coefficient of determination (R^2^) signifies the fraction of variance in observable data accounted for by the model, demonstrating its overall predictive efficacy. The Normalized Mean Square Error (NMSE) offers a comparative assessment of error, facilitating evaluations across datasets with varying dimensions. The Prediction Interval (PI) evaluates the boundaries of uncertainty in forecasts, whereas the Weighted Absolute Percentage Error (WAPE) quantifies relative discrepancies between projected and actual values, providing a normalized view of performance. The relevant equations for each measure are provided below to enable accurate computation and interpretation.

8 $${R}^{2}={\left(\frac{{\sum }_{i=1}^{n}\left({b}_{i}-\overline{b }\right)\left({m}_{i}-\overline{m }\right)}{\sqrt{\left[{\sum }_{i=1}^{n}{\left({b}_{i}-\overline{b }\right)}^{2}\right]\left[{\sum }_{i=1}^{n}{\left({m}_{i}-\overline{m }\right)}^{2}\right]}}\right)}^{2}$$

9 $$RMSE = \sqrt {\frac{1}{n}\mathop \sum \limits_{i = 1}^{n} \left( {m_{i} - b_{i} } \right)^{2} }$$

10 $$NMSE=\frac{1}{n}\sum\limits_{i=1}^{n}\frac{{({m}_{i}-{b}_{i})}^{2}}{{b}_{i}.{m}_{i}}$$

11 $$PI = \pm t \times SE \times \sqrt {\left( {1 + \frac{1}{n} + \frac{{\left( {x^{*} - \overline{x}} \right)^{2} }}{{\Sigma \left( {x_{i} - \overline{x}} \right)^{2} }}} \right)}$$

12 $$WAPE=\mathrm{max}\left[\frac{\left|{b}_{i}-{m}_{i}\right|}{{b}_{i}}\right]$$

Here, $n$ shows the sample number, ${b}_{i}$ shows the predicted value of the target variable for the i-th sample, ${m}_{i}$ shows the observed value of the target variable for the i-th sample, $\overline{m }$ and $\overline{b }$ show the mean value of the observed and predicted values, SE shows the standard error of the regression model, ${x}^{*}$ shows the test input value for which the prediction interval is estimated, ${x}_{i}$ shows the i-th observed input value, and $\overline{x}$ shows the mean of all observed input values.

## Hyperparameter tuning

Table 3 summarizes the optimal hyperparameters identified for both hybrid-optimization–based models and standalone machine learning frameworks used in this study. For CATR-based models (CAAO, CATS, and CAT), several key hyperparameters were explicitly tuned, including tree depth, number of iterations, L2 regularization (l2_leaf_reg), and border_count, which controls the discretization of numerical features. These parameters jointly regulate model complexity, learning stability, and generalization capacity. The depth parameter, which defines the maximum level of tree expansion, was assigned higher values in CAAO compared to CATS and CAT, reflecting the hybrid optimizer’s ability to explore deeper tree structures and capture strong nonlinear interactions among renewable energy system variables. The iterations parameter—representing the number of boosting rounds—was also explicitly configured for all CatBoost-based models, ensuring sufficient learning capacity while avoiding overfitting. Regularization via l2_leaf_reg was applied to constrain excessive model complexity, particularly for deeper trees. In contrast, RFR–based models (RFAO, RFTS, and RFR) rely on ensemble averaging rather than boosting and therefore utilize a different hyperparameter structure. For these models, max_depth, min_samples_leaf, random_state, and n_estimators were tuned to balance tree diversity and ensemble stability. RFAO employed the largest number of estimators, followed by RFTS and RFR, enabling improved variance reduction and predictive robustness.

**Table 3:** The hyperparameters of the hybrid models, along with their assigned values.

Hyperparameter	Hybrid models					
	CAAO	CATS	CAT	RFAO	RFTS	RFR
depth	9	7	4	×	×	×
l2_leaf_reg	7	6	3	×	×	×
border_count	41	50	88	×	×	×
iterations	200	200	20	×	×	×
max_depth	×	×	×	6	8	12
min_samples_leaf	×	×	×	3	4	7
random_state	×	×	×	40	37	22
n_estimators	×	×	×	660	601	550

## Results

This section delineates the study’s results, emphasizing significant trends and observations using tables and figures. The visualizations illustrate the distribution, trends, and correlations within the dataset, providing a clear overview of the investigated characteristics and results. Tables offer succinct numerical summaries, whereas figures depict changes and comparative insights across several settings. Collectively, these data facilitate clear interpretation and discourse, establishing the foundation for comprehending the principal findings. This part establishes a basis for subsequent analysis, directing the interpretation of the data and substantiating the findings derived from the investigation.

### Analysis

**Fig. 8:**
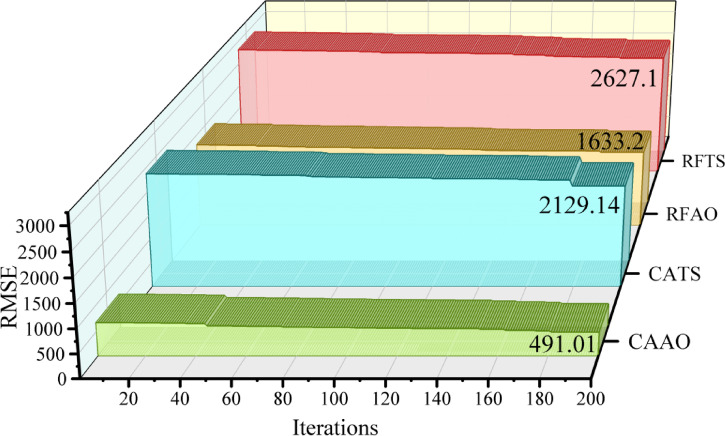
Convergence curve of the four hybrid models based on RMSE and 200 iterations with 3D wall plot.

Figure 8 depicts the convergence characteristics of the optimization-driven frameworks, emphasizing the decrease in prediction error with an increase in the number of iterations. All hybrid models demonstrated incremental enhancements, validating the capacity of optimization processes to improve parameter tuning and increase prediction accuracy in renewable energy emission reduction modeling. CAAO exhibited the most efficient convergence, starting with a comparatively high error that swiftly diminished to a negligible level following adequate repetitions. This signifies robust stability and accelerated learning dynamics relative to alternative frameworks. CATS demonstrated significant error reduction; yet, its convergence trajectory was still superior to that of CAAO, indicating modest predictive efficacy. RFAO achieved a relatively low error level, securing the second position in overall performance, whereas RFTS demonstrated delayed convergence and elevated residual error, indicating suboptimal optimization efficiency. The convergence patterns highlight that optimization techniques enhance learning and directly aid in reducing uncertainty in emission reduction forecasts. Of all frameworks, CAAO demonstrated the most dependable results, affirming its preeminence for precise and consistent forecasting in renewable energy applications.

Table 4 delineates the comparative results of single and hybrid frameworks during the training, validation, and testing stages. Within the singular group, CAT regularly surpassed RFR, exhibiting reduced error levels and a more robust correlation with the objective variable. Nonetheless, both individual models demonstrated somewhat greater deviations and diminished stability when evaluated against hybrid methodologies. The hybrid frameworks showed significant enhancements in prediction performance. CAAO attained the most favorable overall outcomes across all phases, integrating exceptionally low error metrics with nearly flawless accuracy and stability indications. RFAO was identified as the second most successful framework, with significant reductions in error and excellent explanatory power, while CATS also showed great performance with competitive accuracy and modest error rates. Conversely, RFTS, despite improvements over the individual models, fell short of CAAO, RFAO, and CATS in terms of precision and dependability. The persistent dominance of CAAO in training, validation, and testing validates the benefits of optimization-based hybridization. In comparison to singular frameworks, hybrid models not only reduced prediction error but also improved generalization ability. CAAO is the most effective framework, providing strong and highly trustworthy forecasts for the specified application.

**Table 4:** Performance metrics of the models, assessing their predictive accuracy and effectiveness using key statistical indicators.

Process	Framework	Models	Evaluation Metrics				
			RMSE	R^2^	NMSE	PI	WAPE
Train	Single	CAT	3520.5	0.944	1032.8	0.071	0.114
		RFR	3854.4	0.933	1238.0	0.078	0.124
	Hybrid	CAAO	491.0	0.999	20.091	0.010	0.016
		CATS	2191.1	0.977	400.09	0.044	0.071
		RFAO	1633.2	0.987	222.29	0.032	0.053
		RFTS	2627.2	0.968	575.17	0.052	0.085
Validation	Single	CAT	3650.0	0.940	8881.7	0.073	0.119
		RFR	3963.7	0.930	10,474.1	0.080	0.128
	Hybrid	CAAO	507.68	0.999	171.83	0.010	0.016
		CATS	2258.6	0.977	3400.8	0.045	0.073
		RFAO	1624.6	0.988	1759.6	0.032	0.052
		RFTS	2667.5	0.967	4743.6	0.053	0.086
Test	Single	CAT	3534.1	0.943	8326.5	0.071	0.115
		RFR	3901.5	0.932	10,148	0.079	0.128
	Hybrid	CAAO	496.62	0.999	164.42	0.010	0.016
		CATS	2290.6	0.975	3497.9	0.046	0.074
		RFAO	1607.8	0.988	1723.4	0.032	0.052
		RFTS	2653.5	0.966	4694.2	0.053	0.087

**Fig. 9:**
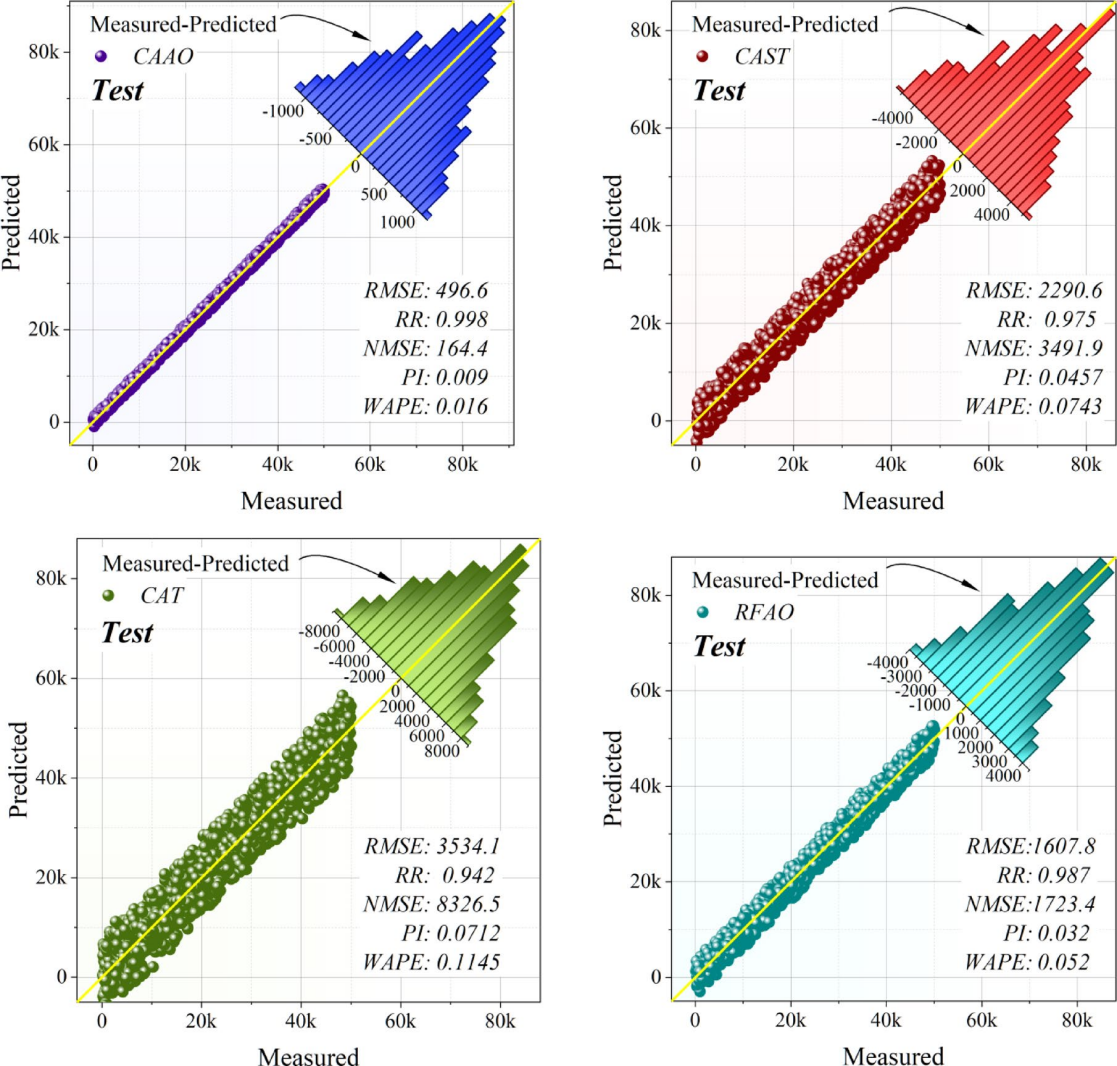

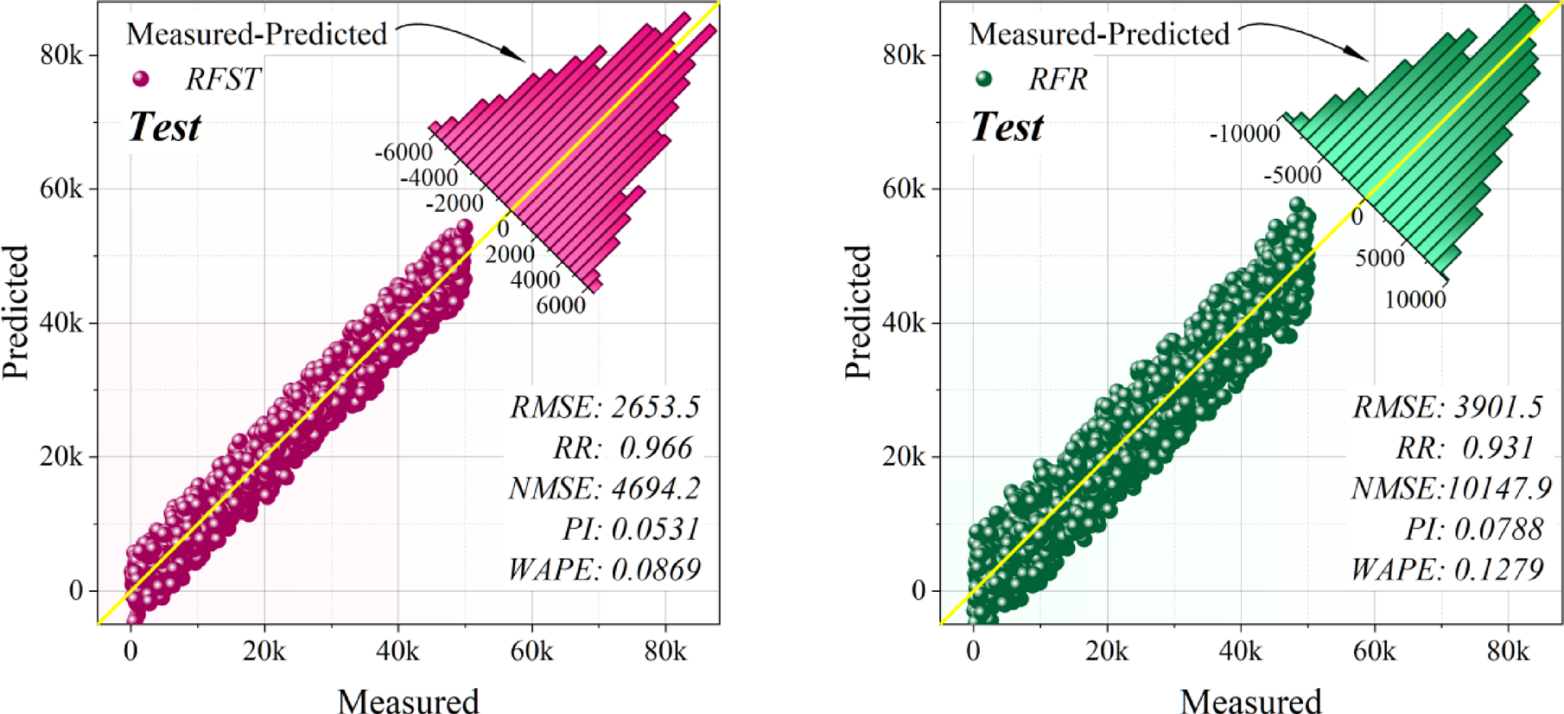
Dispersion of the evolved models.

Figure 9 depicts the dispersion plots of the developed hybrid and singular models, elucidating the correlation between anticipated and actual values for renewable energy emission reduction. The density of points around the best-fit line indicates prediction precision and model dependability. Of all the frameworks, CAAO demonstrated the greatest density around the regression line, proving its exceptional capacity to discern the underlying patterns in the data with low variance. This close grouping underscores its resilience and uniformity over the prediction spectrum. RFAO ranked as the second-best framework, exhibiting robust alignment, but with somewhat more dispersion than CAAO. CATS had adequate prediction accuracy; however, its dispersion pattern was wider, signifying moderate error levels. Conversely, RFTS demonstrated significantly more scattering, indicating worse generalization and less prediction stability. The individual frameworks, CAT and RFR, had the greatest dispersion around the regression line, indicating relatively subpar performance and elevated error magnitudes. The dispersion study indicates that optimization-driven hybridization significantly enhances prediction reliability, with CAAO identified as the most effective framework for precise emission reduction modeling.

**Fig. 10:**
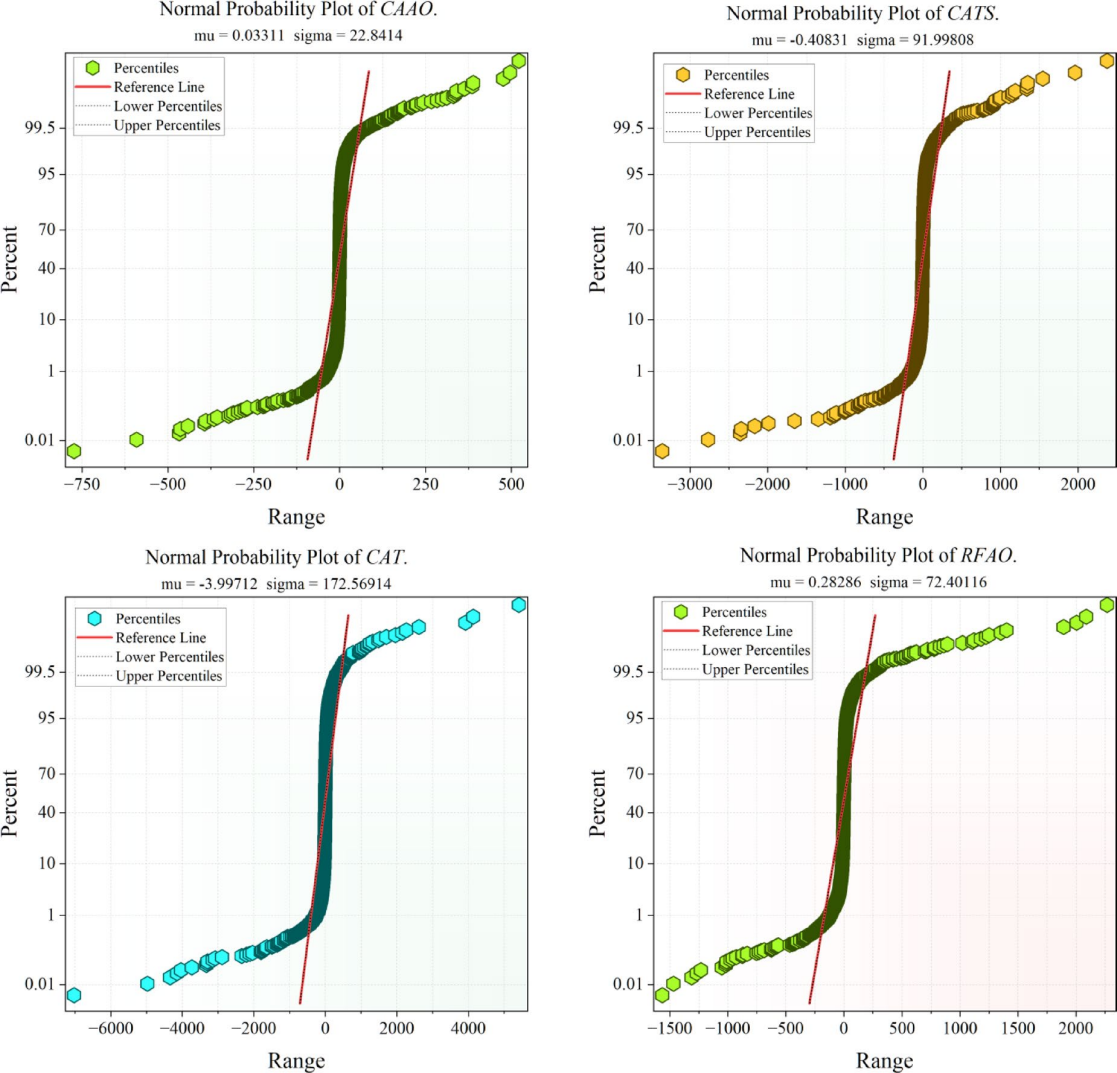

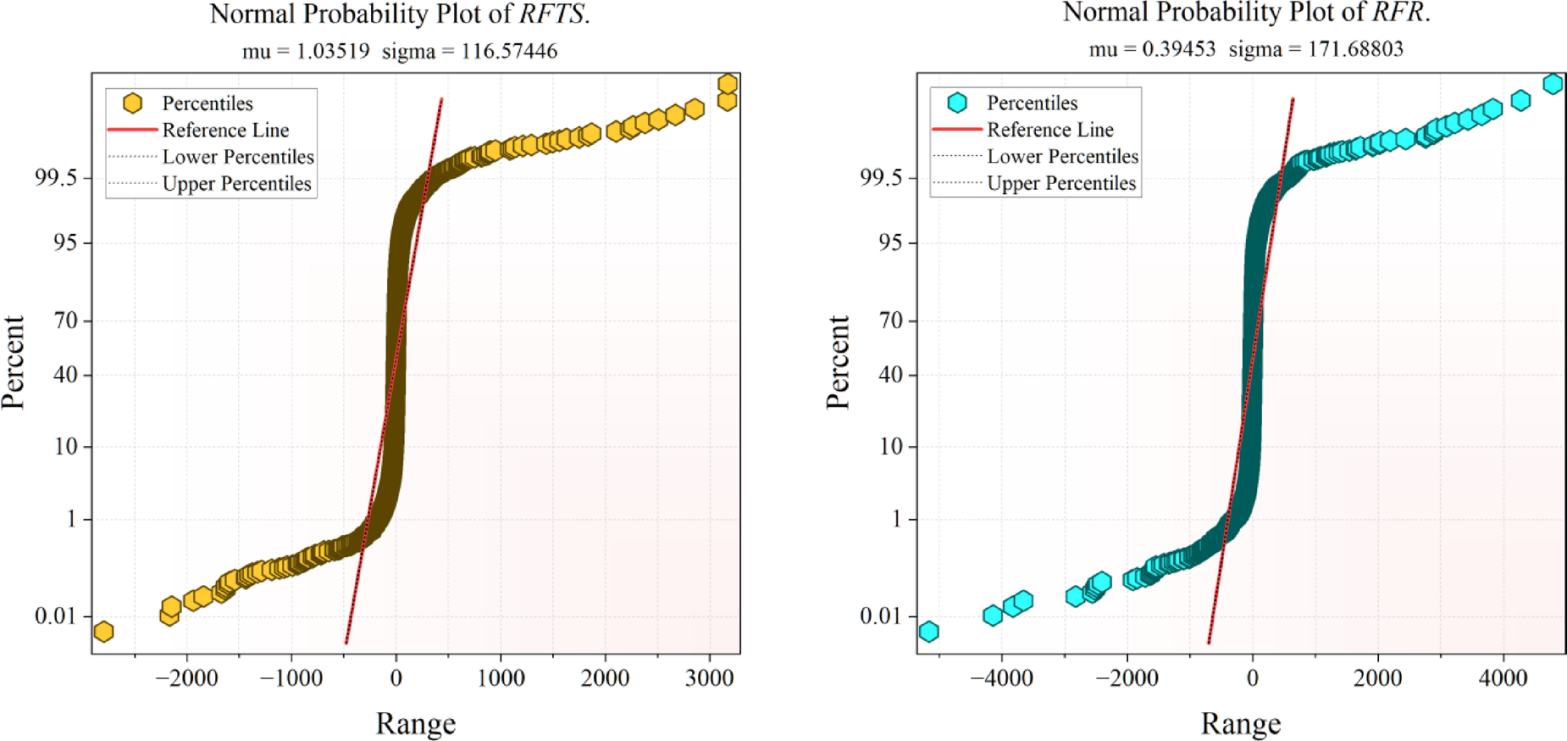
Probability plot errors of the proposed models.

Figure 10 illustrates the probability error maps of the suggested models, offering additional evidence of their predictive accuracy. In accordance with the findings shown in the preceding figures, CAAO once more exhibits superior performance, since its error distribution closely aligns with the optimal reference line. This signifies that the residuals are negligible and symmetrically distributed, demonstrating both precision and consistency in prediction. Consistent with previous results, RFAO emerges as the second most robust model, succeeded by CATS, both demonstrating relatively excellent alignment but exhibiting significantly broader error dispersion in comparison to CAAO. In contrast, RFTS, CAT, and RFR have greater divergences from the reference line, indicating diminished predictive dependability. The probability error plots corroborate the pattern shown in the convergence and dispersion analyses: CAAO routinely surpasses alternative frameworks, affirming its preeminence for precise and resilient modeling of renewable energy emission reduction.

**Fig. 11:**
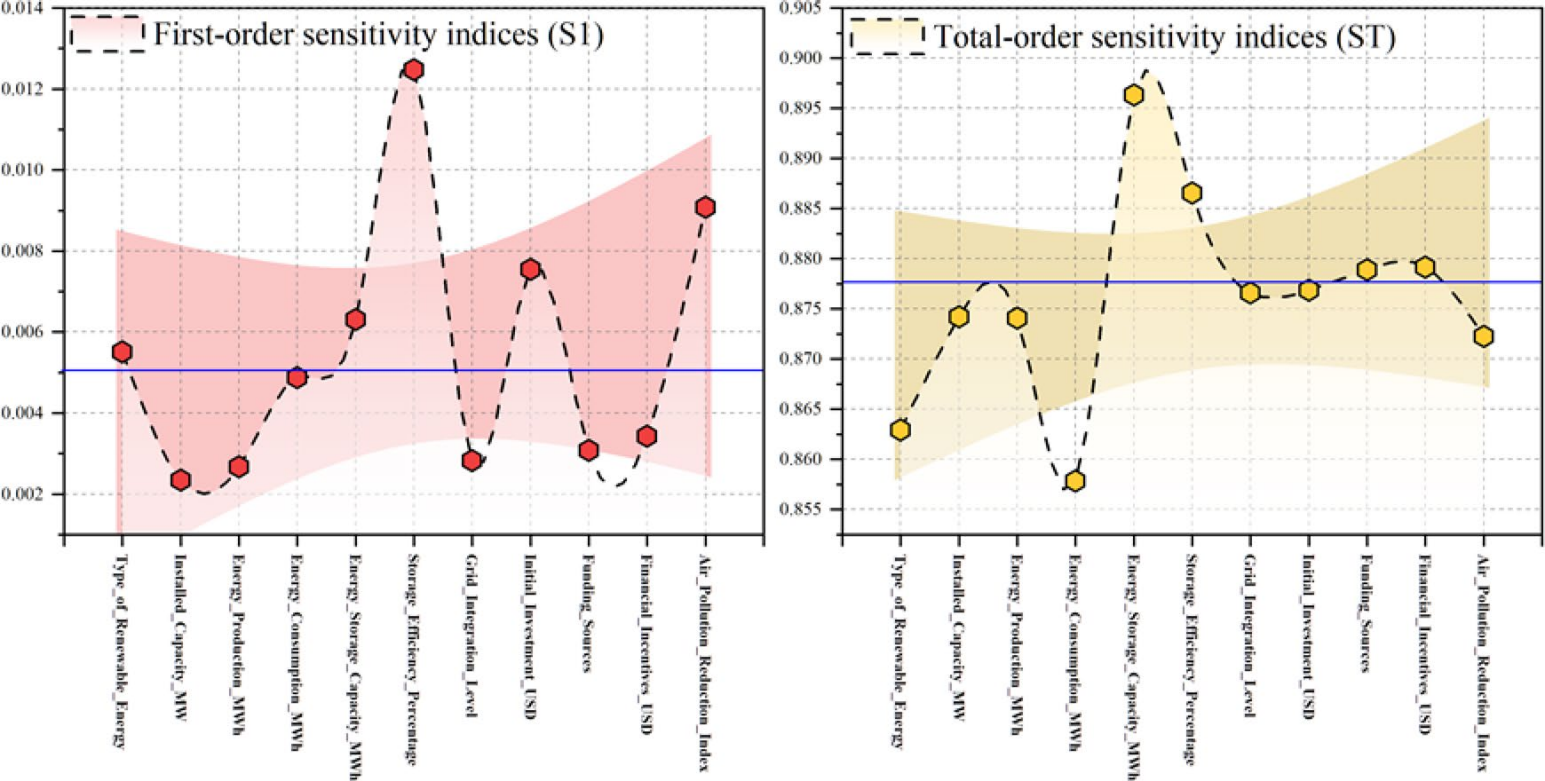
FAST sensitivity analysis for the impact of the input variables on output.

Figure 11 presents a sensitivity analysis that offers a comparative assessment of input variables using both the S1 and ST techniques. The S1 findings, which reflect the direct impact of each element on emission reduction, identify storage efficiency (%) as the paramount determinant. This indicates that optimizing the operating efficiency of storage systems is crucial for augmenting forecasting accuracy. The air pollution reduction index and storage capacity (MWh) are secondary contributors, whereas installed capacity, energy production, and consumption have little impact. The ST approach, which considers both primary effects and higher-order interactions, alters the hierarchy of significance. In this context, storage capacity (MWh) is identified as the predominant element, highlighting the critical need for extensive storage infrastructure in facilitating efficient renewable energy implementation. Storage efficiency (%) is of paramount importance, affirming its impact notwithstanding feature interactions. Economic incentives, grid integration, and environmental indices hold considerable significance; the kind of renewable energy consistently demonstrates the least influence across both methodologies. Collectively, these data suggest that the technical attributes of storage systems, specifically efficiency and capacity, are the primary factors influencing emission reduction, beyond either economic or environmental considerations.

**Fig. 12:**
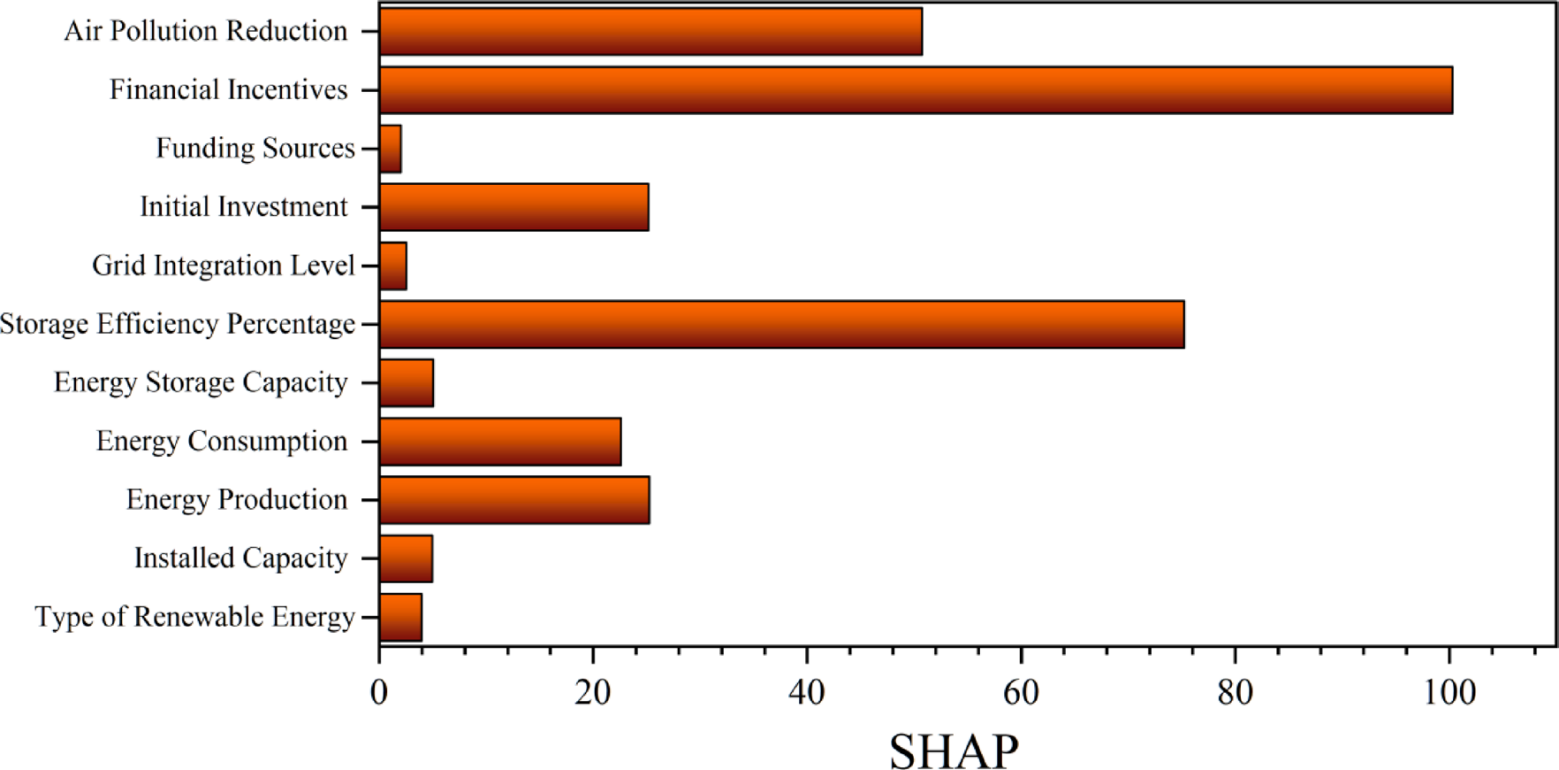
SHAP sensitivity analysis for the impact of the variables on output.

Figure 12 depicts the SHAP sensitivity analysis of the optimal model, offering a comprehensive assessment of feature significance in forecasting renewable energy emission reduction. The research indicates that financial incentives have the most substantial influence, as evidenced by their greatest SHAP values. This discovery underscores the pivotal importance of economic policies and subsidies in expediting the adoption of renewable technology and facilitating quantifiable carbon reductions. The subsequent crucial aspect is the storage efficiency %, underscoring the significance of efficient energy storage systems in improving grid stability and optimizing renewable resource usage. The reduction of air pollution suggests that enhancements in environmental quality are closely correlated with the model’s prediction results. In contrast, financing sources and grid integration have the lowest SHAP values, indicating a diminished impact on predictions relative to other variables. Although these characteristics are significant, their limited contribution underscores that financial and technical processes directly associated with incentives and storage offer superior explanatory power. The SHAP analysis substantiates the preeminence of financial and technological factors in formulating precise emission reduction predictions, consistent with pragmatic Policy and infrastructure objectives.

### Reconciling FAST and SHAP feature rankings

Although both FAST and SHAP are used to assess feature importance, they quantify fundamentally different aspects of model behavior, and therefore their rankings are not expected to coincide exactly. FAST is a global variance-based sensitivity analysis method that decomposes the total output variance into first-order effects (S1) and total effects (ST), the latter capturing higher-order interactions among variables. As such, FAST identifies variables that structurally govern system behavior, particularly through nonlinear interactions and coupled effects. In contrast, SHAP values provide a local, model-specific explanation of predictions by quantifying the average marginal contribution of each feature across all samples. SHAP therefore reflects predictive dominance within the trained model, rather than global system variance. Variables with strong, monotonic effects—such as financial incentives—may exert a large influence on predictions even if they do not dominate interaction-driven variance. The divergence observed in this study is therefore expected: FAST identifies storage capacity and storage efficiency as dominant contributors when interaction effects are considered, highlighting their foundational role in enabling renewable system stability and emission reduction. Conversely, SHAP assigns the highest importance to financial incentives, indicating that policy-driven economic support strongly influences the model’s short-term predictive outcomes. Additionally, correlations among inputs (e.g., between storage deployment, incentives, and environmental indicators) can redistribute importance differently across FAST and SHAP analyses. In such cases, SHAP may attribute shared predictive influence to the most immediately informative variable, while FAST distributes variance across interacting features.

### Feature sensitivity and practical implications

The combined sensitivity analyses demonstrate that storage-related variables and financial incentives influence emission reduction through distinct but complementary mechanisms. FAST analysis reveals that storage efficiency and storage capacity dominate global output variance, particularly when interaction effects are included, underscoring their structural importance in renewable energy system performance. In contrast, SHAP analysis highlights financial incentives as the most influential predictive feature, reflecting their strong and consistent effect on model predictions across samples. This distinction indicates that while storage infrastructure governs the underlying system dynamics and long-term stability, economic incentives primarily drive near-term deployment decisions and observable emission outcomes. Recognizing this methodological difference resolves the apparent discrepancy between FAST and SHAP rankings and strengthens the resulting policy guidance. Effective emission reduction strategies should therefore combine long-term investment in storage capacity and efficiency with adaptive financial incentives to achieve both structural resilience and rapid decarbonization.

## Discussion

The dataset on renewable energy emission reductions exhibits significant diversity across technical, economic, and environmental attributes. Statistical summaries (Table 4) reveal disparities in installed capacity, energy output and consumption, storage capacity, and storage efficiency, highlighting the diversity of renewable energy systems. Economics, including initial investments and payoffs, have large variability, which implies that system deployment may vary based on economic status. Environmental indicators, such as reduction in air pollution and mitigation potential of greenhouse gases, have large variability, which indicates the various results possible under different configurations of a system. The feature-based approach argues that emphasizing specific technological and economic traits would strengthen prediction indicators and mitigation measures. Efficiency and storage capacity are most applicable for optimal energy use and grid stability, while financial incentives would motivate renewable technology diffusion by increasing projects’ economic feasibility in variable conditions. Certain prediction models, including gradient boosting models such as CAT and composite methods RFR, may be applied to investigate nonlinear correlations between such traits. Further, optimization methods such as the AOA and TSOA may be applied to adjust model variables or select feature combinations optimizing prospective emissions reduction. From an application perspective, these methodologies offer direction for formulating renewable energy policies, distributing resources, and assessing the possible effects of initiatives. Constraints encompass data variety, regional distinctiveness, and the necessity to consider dynamic interactions among technological, economic, and environmental factors. Future studies may explore spatiotemporal impacts, multi-criteria optimization, and integrated technical-financial scenarios. The practical ramifications of a feature-driven methodology encompass influencing policy determinations, prioritizing technology advancements, and directing incentive initiatives to potentially augment emission reductions. The research is significant as it illustrates that a systematic assessment of technical, financial, and environmental attributes, along with appropriate predictive and optimization techniques, can provide insights into emission reduction strategies, informing future research and planning (Figs. 11 and 12).

### Limitations

Despite the methodological rigor and comprehensive feature analysis presented in this study, several limitations must be acknowledged. The study uses a Kaggle dataset that provides a synthetic, aggregated approximation of renewable energy systems rather than direct measurements from specific regions. As a result, the dataset may not capture local contextual nuances, and the findings should be interpreted as indicative patterns rather than deterministic predictions. Critical factors such as solar irradiance, wind speed variability, temperature effects, and localized grid constraints are not included. Consequently, the model cannot account for site-specific operational challenges that may influence real-world emission-reduction outcomes. While the dataset includes a wide range of technological, financial, and environmental variables, its lack of location-specific granularity limits the transferability of policy recommendations to specific geographic or climatic settings. To enhance practical applicability, future research will aim to integrate empirical, geographically-resolved datasets, incorporate meteorological data, and validate model predictions under regional operational conditions.

Variables such as financial incentives and funding sources are modeled as static inputs in the current study, based on representative values from the dataset. In reality, these parameters are dynamic and vary over time, influenced by policy revisions, regulatory changes, and market conditions. Consequently, the model may not fully capture the temporal variability and evolving impact of economic instruments on renewable energy adoption and greenhouse gas reductions. Incorporating time-series or scenario-based modeling approaches in future work would improve the fidelity of predictions for real-world policy planning.

Although explicit leakage was not detected, some input variables represent aggregated environmental or economic co-benefits that may share upstream determinants with the target variable. As a result, exceptionally high predictive accuracy may partially reflect structured correlations within the dataset rather than fully independent generalization. This limitation reinforces the exploratory and interpretive scope of the present study. It should be noted that the dataset used in this study does not correspond to a single, explicitly defined geographic or regulatory context. As a result, while the relative importance of factors such as Financial Incentives and Funding Sources is robust within the modeled data distribution, the specific ranking and magnitude of their SHAP values may differ across regions with distinct policy frameworks, market structures, and subsidy mechanisms. Consequently, the presented feature attributions should be interpreted as reflecting generalized structural relationships rather than directly transferable policy priorities for any single national or regional energy system. In regulatory environments where incentive schemes, financing accessibility, or public–private investment mechanisms differ substantially, the dominance of specific financial drivers may shift, underscoring the need for localized recalibration before policy implementation.

Another important limitation pertains to the Air Pollution Reduction Index, which was treated as an input feature in the predictive models. This variable represents a co-outcome of renewable energy deployment rather than a directly manipulable policy or technological lever. Consequently, its high SHAP importance should be interpreted as a proxy for overall system performance and environmental quality, rather than implying that policymakers can directly act on it to reduce greenhouse gas emissions. Future studies aiming at causal policy assessment should distinguish between input drivers and emergent co-outcomes to avoid potential misinterpretation.

### Future research direction

While the present study demonstrates the dominant role of energy storage characteristics and financial mechanisms in predicting greenhouse gas emission reductions, several high-impact research avenues remain open for future investigation.

First, future studies should extend the current focus on storage capacity toward power-to-gas technologies, particularly hydrogen production via electrolysis. Recent research indicates that the spatial allocation of electrolysers, when coordinated with transmission expansion planning, can substantially affect both system costs and emission outcomes. Incorporating electrolyser siting and operation into predictive and optimization frameworks would allow a more comprehensive assessment of sector coupling and long-term decarbonization pathways, especially under high renewable penetration scenarios^33^.

Second, although grid integration is considered in the present work, future research should explicitly model the electrification of the transport sector as a major source of system flexibility. The integration of electric vehicle smart-charging strategies and vehicle-to-grid (V2G) capabilities could be explored as critical assets for mitigating renewable intermittency, alleviating congestion, and enhancing overall grid resilience. Incorporating transport-sector flexibility would enable a more realistic representation of future low-carbon energy systems where electricity, mobility, and storage interact dynamically ^34^.

Third, the observed variability in energy consumption highlights the need for deeper exploration of demand-side management and building-level flexibility. Future investigations could adopt coordinated control strategies for large urban building stocks, leveraging thermal inertia and aggregated space-heating control to provide system-level flexibility. Such approaches have demonstrated strong potential to reduce peak loads, accommodate renewable generation surpluses, and ease operational stress on distribution networks^16^.

Finally, while the current framework applies machine learning and metaheuristic optimization to renewable energy system planning, future work could extend this methodology to renewable fuel supply chains, such as biomass or bioenergy systems. Incorporating location optimization, logistics planning, and risk assessment under uncertainty would allow the evaluation of transport-related emissions and cost trade-offs, further broadening the applicability of data-driven decision-support tools across the energy transition value chain^35^.

Collectively, these directions highlight the potential of combining advanced machine learning, optimization, and sector-coupling concepts to move from predictive assessment toward integrated, system-wide planning solutions for deep decarbonization.

## Conclusion

This study analyzed the prediction of greenhouse gas emission reductions from renewable energy systems by employing a comprehensive set of technical, economic, and environmental factors. Among the models examined, CatBoost Regression (CATR) consistently shows superior prediction accuracy compared to Random Forest Regression (RFR), particularly in identifying the nonlinear relationships inherent in various renewable energy datasets. The hybrid frameworks, including optimization approaches such as the Arithmetic Optimization Algorithm (AOA) and Transit Search Optimization (TSO), markedly enhanced model performance through optimized parameter selection and increased generalization across many scenarios. CAAO emerged as the most effective hybrid approach, exhibiting little error, robust correlation with actual emission reductions, and considerable stability across training, validation, and test datasets. Feature-based analysis indicated that storage-related attributes significantly influence emission reduction projections. A key contribution of this study lies in the combined use of FAST and SHAP sensitivity analyses, which provide complementary yet distinct insights into the drivers of greenhouse gas emission reduction. The FAST analysis reveals the structural importance of energy storage systems, particularly storage capacity, when higher-order interactions are taken into account. This finding indicates that large-scale storage infrastructure plays a fundamental role in stabilizing renewable energy systems and enabling sustained emission reductions through complex interdependencies with generation, grid integration, and operational efficiency. In contrast, the SHAP analysis highlights the predictive importance of financial incentives, demonstrating that economic support mechanisms exert the greatest influence on short-term emission-reduction forecasts. While storage efficiency also remains influential, the dominance of incentives in SHAP reflects their role in accelerating technology adoption and shaping observable deployment outcomes. A key methodological contribution of this study is the explicit reconciliation of sensitivity-based and explainability-based interpretability frameworks. While the FAST analysis identifies energy storage capacity as a dominant structural driver governing the variance of emission reduction outcomes, SHAP analysis highlights financial incentives as the most influential predictive driver shaping model responses across observed scenarios. Rather than representing a contradiction, this divergence reflects complementary perspectives: FAST captures system-level physical influence, whereas SHAP reveals conditional, data-driven importance. This integrated interpretation provides policymakers with a more balanced decision-support perspective than single-method analyses, distinguishing long-term infrastructure leverage from short-term policy effectiveness. Energy storage efficiency is consistently identified as a critical factor, underscoring the imperative to enhance energy storage systems to increase grid flexibility, maximize renewable energy utilization, and augment carbon mitigation capabilities. Financial incentives were identified as a vital component, highlighting the importance of economic Policy in accelerating renewable adoption and aiding carbon reduction initiatives. The interaction of technical and financial factors suggests that combined approaches focusing on both system efficiency and investment frameworks may be most effective in reducing emissions. The findings indicate that storage efficiency-focused predictive modeling, employing state-of-the-art regression and hybrid optimization methods, provides meaningful insights for policymakers, investors, and system planners. By identifying the most important attributes and employing stringent modeling strategies, stakeholders can focus efforts, boost renewable energy installations, and enable targeted carbon-reduction programs. The study demonstrates feature-focused predictive analysis as a robust methodological framework for investigating large multivariate systems, yielding a scalable platform for evaluating renewable energy installations across divergent geographical and operational configurations.

## Data Availability

Data can be obtained from the corresponding author upon reasonable request (subashsubashch12@gmail.com).

## References

[1] Wang, J. & Azam, W. Natural resource scarcity, fossil fuel energy consumption, and total greenhouse gas emissions in top emitting countries. *Geosci. Front.* **15**(2), 101757 (2024).

[2] Patil, G., Pode, G., Diouf, B. & Pode, R. Sustainable decarbonization of road transport: Policies, current status, and challenges of electric vehicles. *Sustainability* **16**(18), 8058 (2024).

[3] Jha, M. K. & Dev, M. Impacts of climate change. In *Smart Internet of Things for Environment and Healthcare* 139–159 (Springer, 2024).

[4] Hariram, N. P., Mekha, K. B., Suganthan, V. & Sudhakar, K. Sustainalism: An integrated socio-economic-environmental model to address sustainable development and sustainability. *Sustainability* **15**(13), 10682 (2023).

[5] Anser, M. K., Khan, K. A., Umar, M., Awosusi, A. A. & Shamansurova, Z. Formulating sustainable development policy for a developed nation: Exploring the role of renewable energy, natural gas efficiency and oil efficiency towards decarbonization. *Int. J. Sustain. Dev. World Ecol.* **31**(3), 247–263 (2024).

[6] Yang, H.-C., Feng, G.-F., Zhao, X. X. & Chang, C.-P. The impacts of energy insecurity on green innovation: A multi-country study. *Econ. Anal. Policy* **74**, 139–154 (2022).

[7] Yin, H.-T., Chang, C.-P. & Wang, H. The impact of monetary policy on green innovation: Global evidence. *Technol. Econ. Dev. Econ.* **28**(6), 1933–1953 (2022).

[8] Islam, M. M. et al. Improving reliability and stability of the power systems: A comprehensive review on the role of energy storage systems to enhance flexibility. *IEEE Access* **12**, 152738–152765 (2024).

[9] Raihan, A. & Tuspekova, A. The nexus between economic growth, renewable energy use, agricultural land expansion, and carbon emissions: New insights from Peru. *Energy Nexus* **6**, 100067 (2022).

[10] Raihan, A. & Voumik, L. C. Carbon emission dynamics in India due to financial development, renewable energy utilization, technological innovation, economic growth, and urbanization. *J. Environ. Sci. Econ.* **1**(4), 36–50 (2022).

[11] Saleh, H. M. & Hassan, A. I. The challenges of sustainable energy transition: A focus on renewable energy. *Appl. Chem. Eng.* **7**(2), 2084 (2024).

[12] Posacka, K. Reducing CO_2_ emissions into the atmosphere using the main engine settings of maritime vessels. *Zesz. Nauk. Politech. Morskiej w Szczecinie* (2024).

[13] Lee, H. et al. Climate change 2023 synthesis report summary for policymakers. In *Clim. Chang. 2023 Synth. Rep. Summ. Policymakers* (2024).

[14] Zhou, T., Ma, Z., Wen, Q., Wang, X., Sun, L. & Jin, R. Fedformer: Frequency enhanced decomposed transformer for long-term series forecasting. In *International Conference on Machine Learning*, 27268–27286 (2022).

[15] Giannelos, S., Pudjianto, D., Zhang, T. & Strbac, G. Energy hub operation under uncertainty: Monte Carlo risk assessment using gaussian and KDE-based data. *Energies* **18**(7), 1712 (2025).

[16] Dong, Z., Zhang, X., Zhang, L., Giannelos, S. & Strbac, G. Flexibility enhancement of urban energy systems through coordinated space heating aggregation of numerous buildings. *Appl. Energy* **374**, 123971 (2024).

[17] Giannelos, S., Konstantelos, I., Zhang, X. & Strbac, G. A stochastic optimization model for network expansion planning under exogenous and endogenous uncertainty. *Electr. Power Syst. Res.* **248**, 111894 (2025).

[18] Du, Z. et al. Decarbonisation of data centre networks through computing power migration. In *2025 IEEE 5th International Conference on Computer Communication and Artificial Intelligence (CCAI)*, 871–876 (2025).

[19] Wang, Y., Li, K. & Peng, L. Revesting the spatial spillover effects of renewable energy in curbing carbon emissions: Evidence from China. *Energy Environ.*10.1177/0958305X241296447 (2024).

[20] Abidi, I. & Nsaibi, M. Assessing the impact of renewable energy in mitigating climate change: A comprehensive study on effectiveness and adaptation support. *Int. J. Energy Econ. Policy* **14**(3), 442–454 (2024).

[21] Heshmati, A., Abolhosseini, S. & Altmann, J. Impact of renewable energy development on carbon dioxide emission reduction. In *The Development of Renewable Energy Sources and its Significance for the Environment* 119–146 (Springer, 2015).

[22] Kumi, E. N. & Mahama, M. Greenhouse gas (GHG) emissions reduction in the electricity sector: Implications of increasing renewable energy penetration in Ghana’s electricity generation mix. *Sci. Afr.* **21**, e01843 (2023).

[23] Dulal, A. et al. Impact of renewable energy on carbon dioxide emission reduction in Bangladesh. *J. Power Energy Eng.* **9**(5), 134–165 (2021).

[24] Kurte, K. R., Raju, M. M., Dongritot, P. & Kulkarni, K. Budget-constrained Emission Reduction in Economic and Environmental Dispatch. In *2023 IEEE International Conference on Power Electronics, Smart Grid, and Renewable Energy (PESGRE)*, 1–6 (2023).

[25] Marouani, I. Contribution of renewable energy technologies in combating phenomenon of global warming and minimizing GHG emissions. *Clean Energy Sci. Technol.* **2**(2), 164 (2024).

[26] Breiman, L., Friedman, J., Olshen, R. & Stone, C. *Classification and Regression Trees* (CRC Press, 1984).

[27] Biau, G. & Scornet, E. A random forest guided tour. *TEST* **25**, 197–227 (2016).

[28] Prokhorenkova, L., Gusev, G. Vorobev, A., Dorogush, A. V. & Gulin, A. CatBoost: Unbiased boosting with categorical features. In *Advances in Neural Information Processing Systems*, Vol. 31 (2018).

[29] Dorogush, A. V., Ershov, V. & Gulin, A. CatBoost: Gradient boosting with categorical features support. arXiv Prepr. http://arxiv.org/abs/1810.11363 (2018).

[30] Hashim, F. A., Hussain, K., Houssein, E. H., Mabrouk, M. S. & Al-Atabany, W. Archimedes optimization algorithm: A new metaheuristic algorithm for solving optimization problems. *Appl. Intell.* **51**(3), 1531–1551 (2021).

[31] Qais, M. H., Hasanien, H. M. & Alghuwainem, S. Transient search optimization for electrical parameters estimation of photovoltaic module based on datasheet values. *Energy Convers. Manage.* **214**, 112904 (2020).

[32] Mohamed, S. Tawfik, R. M., Elbayoumi, M. & Darweesh, M. S. Efficient UAV-aided data acquisition based on transit search optimization algorithm. In *2023 5th Novel Intelligent and Leading Emerging Sciences Conference (NILES)*, 184–187 (2023).

[33] Giannelos, S., Konstantelos, I., Pudjianto, D. & Strbac, G. The impact of electrolyser allocation on Great Britain’s electricity transmission system in 2050. *Int. J. Hydrogen Energy* **202**, 153097 (2026).

[34] Amann, G. et al. E-mobility deployment and impact on grids: impact of EV and charging infrastructure on European T&D grids: innovation needs (2022).

[35] Giannelos, S., Konstantelos, I. & Strbac, G. Optimal supply chain design using machine learning, risk assessment and optimisation applied to coal distribution. *EURO J. Decis. Process.*10.1016/j.ejdp.2025.100062 (2025).

